# Spatially-resolved metabolic cooperativity within dense bacterial colonies

**DOI:** 10.1186/s12918-015-0155-1

**Published:** 2015-03-18

**Authors:** John A Cole, Lars Kohler, Jamila Hedhli, Zaida Luthey-Schulten

**Affiliations:** Department of Physics, University of Illinois, 1110 W. Green St., Urbana, 61801 IL USA; Department of Chemistry, University of Illinois, 600 S. Matthews Ave., Urbana, 61801 IL USA; Department of Bioengineering, University of Illinois, 1304 W. Springfield Ave., Urbana, 61801 IL USA

**Keywords:** Flux balance analysis, Reaction-diffusion modeling, Metabolic cooperativity, Crossfeeding, Colony modeling

## Abstract

**Background:**

The exchange of metabolites and the reprogramming of metabolism in response to shifting microenvironmental conditions can drive subpopulations of cells within colonies toward divergent behaviors. Understanding the interactions of these subpopulations—their potential for competition as well as cooperation—requires both a metabolic model capable of accounting for a wide range of environmental conditions, and a detailed dynamic description of the cells’ shared extracellular space.

**Results:**

Here we show that a cell’s position within an *in silicoEscherichia coli* colony grown on glucose minimal agar can drastically affect its metabolism: “pioneer” cells at the outer edge engage in rapid growth that expands the colony, while dormant cells in the interior separate two spatially distinct subpopulations linked by a cooperative form of acetate crossfeeding that has so far gone unnoticed. Our hybrid simulation technique integrates 3D reaction-diffusion modeling with genome-scale flux balance analysis (FBA) to describe the position-dependent metabolism and growth of cells within a colony. Our results are supported by imaging experiments involving strains of fluorescently-labeled *E. coli*. The spatial patterns of fluorescence within these experimental colonies identify cells with upregulated genes associated with acetate crossfeeding and are in excellent agreement with the predictions. Furthermore, the height-to-width ratios of both the experimental and simulated colonies are in good agreement over a growth period of 48 hours.

**Conclusions:**

Our modeling paradigm can accurately reproduce a number of known features of *E. coli* colony growth, as well as predict a novel one that had until now gone unrecognized. The acetate crossfeeding we see has a direct analogue in a form of lactate crossfeeding observed in certain forms of cancer, and we anticipate future application of our methodology to models of tissues and tumors.

**Electronic supplementary material:**

The online version of this article (doi:10.1186/s12918-015-0155-1) contains supplementary material, which is available to authorized users.

## Background

A cell’s metabolic behavior is tightly coupled to its local microenvironment; with it the cell exchanges both food and waste, and from it the cell detects useful information such as shifts in substrate availability. Cells have evolved complex biochemical networks in order to respond to environmental changes, including regulatory systems that enable them to feed on a diverse range of substrates. For diffuse populations living in well-stirred conditions the depletion of food and accumulation of metabolic waste can be slow and spatially uniform, meaning that the behavior of any given cell is largely independent of the others. In contrast, within a colony the close proximity of nearby cells competing for the same diffusing resources can create steep chemical gradients capable of significantly altering each cell’s metabolism. Under these conditions, the behavior of neighboring cells can be strongly interdependent [1], and understanding their interactions requires a detailed picture of both the shared environment and the cells’ metabolic responses to it.

Several approaches have been developed in the past to analyze the intercellular interactions of large numbers of microbes in close spatial proximity (for a review, see [2]). In general, these models have employed highly simplified kinetic descriptions of nutrient uptake and cell growth. Despite the numerous successes of these methods, oversimplification—especially of complex cellular networks like metabolism—can fail to capture important collective behavior. *E. coli* metabolism alone involves thousands of reacting substrates and enzymes, and while many individual metabolic pathways are well characterized, understanding how these pathways interact on a systems level remains a challenge. Flux balance analysis (FBA) [3,4], which uses linear programming techniques to find the set of reaction fluxes that optimize growth, has proven to be a powerful tool for investigating the genome-scale metabolism of bacteria and other organisms under different environmental conditions and in different gene-expression states [5,6]. Recently, a method using FBA in both a spatially- and temporally-resolved manner was described in [7]. This approach made iterative use of the GPU-accelerated Lattice Microbes software [8] to model the diffusion of substrates throughout a cluster of fixed cells, and FBA to model each individual cell’s metabolism. While refinements to the method predicted the emergence of a large region of anaerobically-growing cells within a modeled *E. coli* colony and significant acetate production [9,10], the single molecule resolution of the method made it better suited to studying the interactions of a small number of cells (∼100) in low concentrations of metabolites.

**Figure 1:**
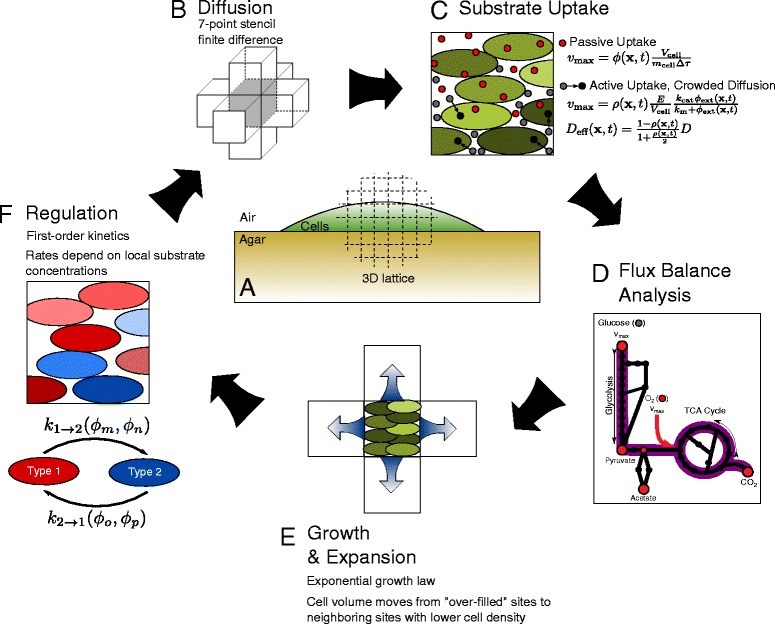
**s3DdFBA methodology at a glance.** **(A)** Cells, agar, and air are discretized to a 3D cubic lattice. **(B)** Substrate diffusion is accounted for using a seven-point stencil finite difference scheme. **(C)** Substrates can be passively or actively taken up by the cells. Those that cannot passively penetrate cell membranes experience hindered diffusion around cells in the extracellular space **(D)** Flux balance analysis predicts substrate usage and cell growth. **(E)** Cell volume grows exponentially until it surpasses a maximum volume fraction, *ρ*_max_, at which time intercellular forces create pressure that pushes cell volume outward into neighboring lattice sites of lesser volume fraction. **(F)** Cells of different species or in different regulatory states can be simultaneously simulated. Those in different states can transform back and forth at rates that can depend on up to two local substrate concentrations, (*ϕ*_m_ and *ϕ*_n_, or *ϕ*_o_ and *ϕ*_p_).

In order to simulate larger and denser colonies over long timescales with higher metabolite concentrations, we have developed a coarse-grained method in which both cell density and substrate concentrations are discretized to a cubic lattice. We model the 3D diffusion, uptake, and efflux of substrates within and around a growing colony of *E. coli* (see Figure 1) by coupling a reaction-diffusion simulation with a genome-scale flux balance metabolic model. This technique, which we call **3DdFBA** (**3-D**imensional **d**ynamic **F**lux **B**alance **A**nalysis), offers powerful insight into how spatial localization within microbial colonies can impact metabolism at the level of individual pathways and reactions. Our simulations reveal how steep glucose and oxygen gradients emerge within the modeled colonies and give rise to four well-defined metabolic phenotypes—a fast-growing ring of cells near the edge making use of the TCA cycle and electron transport chain, a large region of nearly dormant cells in the colony interior, and a pair of spatially distinct crossfeeding subpopulations comprised of acetate-producing fermentative cells near the colony base and acetate-consuming cells higher up. Imaging experiments involving fluorescently labeled *E. coli* strains strongly support these predictions. We also find that the spatial distribution of growth rates within the simulated colonies lead to 3D cross-sections and a linear radial expansion that agree with experimental results.

## Results and discussion

We simulated 48 hours of *E. coli* colony growth on an agar plate containing M9 minimal medium supplemented with 2.5 g l ^−1^ glucose and trace elements. The *E. coli* K-12 MG1655 strain was modeled using the *iJO1366* metabolic reconstruction [4]. The simulations were initialized with the equivalent volume fraction of a single cell in the center of an approximately 3.2 × 3.2 mm agar surface of depth approximately 1 mm. Oxygen was allowed to diffuse into the colony directly from the air as well as through the agar, while glucose was allowed diffuse through the agar alone. The M9 salts and trace elements were not assumed to be growth-limiting, and so their concentrations were not modeled explicitly in the 3D spatiotemporal simulation, but they were allowed to be freely taken up by the cells of the colony. Expecting significant fermentation on the interior of the growing colony [10], preliminary FBA calculations were performed in order to anticipate which fermentative products may play an important role in the cells’ collective metabolism. Formate, acetate, and ethanol were all predicted to be produced in significant amounts, with formate being produced at roughly twice the rate of acetate and ethanol. Succinate was predicted to be produced at roughly 1% of the acetate production rate, while lactate was not predicted to be produced by the modeled cells at all. Because neither formate nor ethanol are used by wild-type *E. coli* as a carbon source and because succinate was produced in such small quantities, the only fermentation product that was ultimately tracked in the spatiotemporal model was acetate. Dirichlet boundary conditions were imposed for the simulated oxygen, glucose, and acetate. The glucose concentration on the walls and floor of the agar was held fixed at 2.5 g l ^−1^, while the oxygen concentration on all boundaries was fixed at 260 *μ*M (the dissolved concentration of oxygen in water under standard laboratory conditions, chosen to approximate the adsorbtion of oxygen as a purely diffusive process). The acetate concentration on the boundaries was fixed at 0.0, ensuring that all acetate within the simulation was created by the cells themselves. There are no free parameters in our simulations—all are either taken from the literature or fit to experimental results. All parameters used in our calculations are summarized in Table 1.

**Table 1:** **Parameters used in our 3DdFBA simulations**

**Parameter**	**Description**	**Value**	**Units**	**Reference or rationale**
*D*_glc, aq_	Diffusion coefficient of glucose in water	7.8×10^−10^	*m* ^2^ *s* ^−1^	[11]
$\phantom {\dot {i}\!}D_{\text {O}_{2}\text {, aq}}$	Diffusion coefficient of O _2_ in water	2.6×10^−9^	*m* ^2^ *s* ^−1^	[11]
*D*_ace, aq_	Diffusion coefficient of acetate in water	1.2×10^−9^	*m* ^2^ *s* ^−1^	[12]
*D*_glc, agar_	Diffusion coefficient of glucose in 1.5% agar	7.4×10^−10^	*m* ^2^ *s* ^−1^	[11]
$\phantom {\dot {i}\!}D_{\text {O}_{2}\text {, agar}}$	Diffusion coefficient of O _2_ in 1.5% agar	2.5×10^−9^	*m* ^2^ *s* ^−1^	[11]
*D*_ace, agar_	Diffusion coefficient of acetate in 1.5% agar	1.1×10^−9^	*m* ^2^ *s* ^−1^	[11,12]
*λ*	Lattice spacing	10.0	*μ* *m*	*λ*<40 *μ*m, the experimental O _2_ penetration depth ([13])
*L*_x_	Simulation volume x dimension	3.2	*mm*	
*L*_y_	Simulation volume y dimension	3.2	*mm*	
*L*_z_	Simulation volume z dimension	1.92	*mm*	
*H*_agar_	Agar height	0.96	*mm*	
*Δ* *τ*	Diffusion, Active Substrate Uptake, FBA time step	1×10^−3^	*s*	$\phantom {\dot {i}\!}\Delta \tau \ll \frac {\lambda ^{2}}{2 D_{\text {O}_{2}\text {, aq}}}$
*t*_ss_	Concentration profile steady state relaxation time	1	*s*	see Expanded View Section 1
*t*_grow_	Time between growth events	60	*s*	see Expanded View Section 1
[*O*_2_]_air_	O _2_ concentration in the air	2.6×10^−4^	*M*	(computed assuming Henry’s law [14])
[*O*_2_]_agar, boundary_	O _2_ concentration fixed on the boundary of the agar	2.6×10^−4^	*M*	(assuming agar and air in equilibrium)
[glc]_agar, boundary_	Glucose concentration fixed on the boundary of the agar	1.39×10^−2^	*M*	
*ρ*_max_	Maximum volume fraction of cells within a colony	0.65	dimensionless	[15]
*Δ* *ρ*	Colony expansion cutoff	0.01	dimensionless	
*m*_cell_	Mass of a single *E. coli* cell	2.58×10^−13^	*g*	[6]
*V*_cell_	Volume of a single *E. coli* cell	1×10^−18^	*m* ^3^	[6]
*v*_max, glc uptake_	Maximum uptake rate for glucose	10.4	mmol gDwt ^−1^ hr ^−1^	fit to data in [16]
*k*_m, glc uptake_	Michaelis constant for glucose uptake	0.37	*mM*	fit to data in [16]
*v*_max, ace uptake_	Maximum uptake rate for acetate	16.0	mmol gDwt ^−1^ hr ^−1^	[17]
$\phantom {\dot {i}\!}v_{\text {max, O\(_{2}\) uptake}}$	Maximum uptake rate for oxygen	31.8	mmol gDwt ^−1^ hr ^−1^	(rate required to aerobically metabolize
				glc and ace at their max uptake rates)

### 3DdFBA methodology

A major result of this study was the development of the 3DdFBA methodology used to perform the simulations; it is outlined in the pseudocode provided in Algorithm 1 and in Figure 1. Both the colony and its surrounding environment are discretized to a 3D lattice (see Figure 1A). Chemical species—represented by a lattice of local concentrations—can diffuse throughout the simulation volume and be taken up or produced by the cells of the colony. Within the colony, the number of cells in a given lattice site is expressed in terms of a local volume fraction, *ρ*, which can range continuously up to some user-defined *ρ*_max_. This *ρ*_max_ represents the maximum cell volume fraction capable of being packed into a given lattice site before intercellular forces begin to shove some of the cells outward into neighboring sites. FBA is used in order to predict how the cells in each lattice site will respond to the concentrations of substrates available to them—they may take up some of one substrate and produce some of another, and grow at some rate as a result. These predictions are used to update the local substrate concentrations and the local cell volume fractions. Cells are allowed to be of different species and/or in different regulatory states. These different cell types or states may use different flux balance models or the same model but with different constraints imposed (when, for example, simulating cells with certain genes “knocked-out”).

Our implementation exploits a natural time-scale separation between the rate at which small molecules like glucose or oxygen diffuse and the rate at which a colony grows. Because the physical size, shape, and regulatory state of a colony changes relatively slowly, fairly long time steps, *t*_grow_, can be safely taken between updates. These time steps can be on the order of minutes (for fast-growing microbes like *E. coli*) to hours (for slow-growing microbes like *Methanosarcina acetivorans* [18]). Substrate concentration profiles, on the other hand, can approach steady state in as short as a few seconds or less. This means that these concentration profiles always remain effectively at steady state with respect to the growth of the colony. This is important for two reasons. First, the assumption that FBA is a valid description of the behavior of a cell hinges on the cell and its environment being at steady state. Second, and more practical, it allows for significant simulation speedup. Simulation of the diffusion, uptake, and utilization of substrates can be performed for relatively short times, *t*_ss_≪*t*_grow_, until they come to steady state, and the results can be used to project forward until the next colony size update. Our own simulations used values of *t*_grow_=60 s and *t*_ss_=1 s in order to ensure moderate colony growth between updates and ample time for the concentration profiles of all simulated chemical species to relax to steady state(see Table 1 and Additional file 1: Section 1.1).

Substrate profiles are brought to steady state through the iterative application of the code’s Diffusion, Active Substrate Uptake, and FBA kernels (see below for details). The Diffusion kernel employs a seven-point stencil finite-difference scheme to account for the diffusion of substrates between lattice sites (see Figure 1B). The Active Substrates Uptake kernel allows for substrates known to be actively imported by the cells of each lattice site to be taken up in accordance with assumed Michaelis-Menten kinetics (see Figure 1C). Finally the FBA kernel is used to predict how much of each substrate the cells of each lattice site produce or consume, as well as those cells’ average growth rate (see Figure 1D). Steady state FBA solutions have been used in the past in iterative time-stepping ways similar to this [19-21], but never with the full 3D spatial resolution described here.

Once brought to steady state, the substrate concentrations and cellular growth rates are used in subsequent colony size and regulatory state calculations. This is done by application of the Growth, Expansion, and Regulation kernels (again, see below for details). The Growth kernel updates the values of the local volume fraction *ρ* within the colony in accordance with an exponential growth law and the local growth rates predicted by FBA. Then, in the event that some lattice sites contain values of *ρ* greater than *ρ*_max_, the Expansion kernel iteratively redistributes some of these sites’ excess volume fractions to neighboring sites with lesser volume fractions until every site falls within a small cutoff of *ρ*_max_. This process effectively expands the colony (see Figure 1E) and ensures that the cell density and intercellular pressure remain relaxed throughout the colony. Finally, the Regulation kernel updates the regulatory states of the cells in each lattice site in accordance with assumed first-order kinetics. Because the cells’ regulatory state can be strongly influenced by environmental factors, the local rates at which they change are assumed to be functions of the local substrate concentrations (see Figure 1F).



#### Diffusion kernel

Each lattice site in the simulation volume is specified as one of three types: air-type, agar-type, or cell-type (see Figure 1A). The air-type lattice sites behave essentially as a source for gaseous substrates like oxygen. The concentration of a substrate in an air-type site is fixed at the concentration of the dissolved gas in water at standard laboratory temperature and pressure. This choice approximates the adsorption of particles from air to agar (or cells) as a simple diffusive process. In effect, it is assumed that right at the boundary between the air and the agar or cells there exists a thin film of water that remains at equilibrium (in terms of the forward and reverse adsorbtion reactions) with the air above it. The oxygen concentration in the air-type sites was computed using Henry’s law to be 260 *μ*M (assuming the partial pressure of O _2_ to be 0.2 atm and a Henry’s law constant of 0.0013 mol l ^−1^ atm ^−1^ [14]). Diffusive flux is allowed from the air-type sites into the agar- and cell-type sites, but it is disallowed from the cells and agar into the air, ensuring that aqueous substrates like glucose and acetate cannot escape. The cell- and agar-type lattice sites are allowed to diffusively exchange substrates, but their local diffusion coefficients differ. In agar, substrates diffuse at rates taken from the literature, which for glucose and oxygen in 1.5% agar, are approximately 95% of their diffusion rates in water (see Table 1 and [11]). The diffusion rates of substrates in cell-type sites depend on the local cell volume fraction and the substrate. Oxygen, for example, readily diffuses through cell membranes [22], and is assumed to diffuse at a similar rate through cells as it does through water. Conversely, glucose, which cannot diffuse passively through the cell membrane [23], is assumed to have to diffuse around cells, and thus experiences a crowded environment and correspondingly slowed diffusion (see Figure 1C). The effective diffusion rates of these hindered substrates are given approximately by [24]:

(1) $$  D_{\text{eff}}(\mathbf{x}, t) = \frac{1 - \rho(\mathbf{x}, t)}{1 + \frac{\rho(\mathbf{x}, t)}{2}} D  $$

where *D* is the diffusion rate of the substrate in water and *ρ*(**x**,*t*) is the instantaneous local cell volume fraction at lattice site **x**.

Among the cell- and agar-type lattice sites, diffusion is modeled using a seven-point stencil finite difference approach. The extracellular concentration of a substrate *ϕ* in site *i* is updated as:

(2) $$  \phi_{i}(\tau + \Delta\tau) = \phi_{i}(\tau) + \frac{\Delta\tau}{\lambda} \sum_{j} J_{\phi, j \rightarrow i}(\tau)  $$

where *λ* is the lattice spacing, *j* indexes over the 6 lattice sites neighboring site *i*, and *J*_*ϕ*,*j*→*i*_ represents the diffusive flux across the boundary seperating sites *j* and *i*. This flux is computed as:

(3) $$  J_{\phi, j \rightarrow i}(\tau) = \frac{D_{\phi, j}(\tau) + D_{\phi, i}(\tau)}{2} \frac{\phi_{j}(\tau) - \phi_{i}(\tau)}{\lambda}  $$

where *D*_*ϕ*,*i*_ and *D*_*ϕ*,*j*_ are the diffusion coefficients for the substrate in sites *i* and *j*, respectively. Here it is important to note that we have averaged the diffusion coefficients for sites *i* and *j* rather than using one or the other; this helps to ensure continuity when *D*_*ϕ*,*i*_≠*D*_*ϕ*,*j*_.

A lattice spacing of 10 *μ*m was used in our simulations. This was chosen in order to resolve the oxygen profile within the colony which is known to fall to nearly zero within approximately 40 *μ*m from the surface [13]. This, coupled with the diffusion rate of oxygen—the fastest diffusing species in the simulation—set a maximum theoretical value for $\Delta \tau \leq \frac {\lambda ^{2}}{2 D_{\text {O\(_{2}\)}}} \approx 2 \times 10^{-2}$, although a more conservative value of 1×10^−3^ s was used in order to ensure convergence.

#### Active substrate uptake kernel

As the cells of the colony actively import glucose, the local extracellular and intracellular glucose concentrations change. This process is assumed to be governed by a Michaelis-Menten-type chemical reaction (see Figure 1C):

(4) $$ \begin{aligned} \phi_{\text{ext}}(\mathbf{x}, t + \Delta\tau) &= \phi_{\text{ext}}(\mathbf{x}, t) - k(\mathbf{x}, t) \Delta\tau \\ \phi_{\text{int}}(\mathbf{x}, t + \Delta\tau) &= \phi_{\text{int}}(\mathbf{x}, t) + k(\mathbf{x}, t) \Delta\tau \end{aligned}  $$

where *ϕ*_ext_(**x**,*t*) and *ϕ*_int_(**x**,*t*) are the instantaneous local concentrations of the extracellular and intracellular forms, *Δτ* is the time step, and *k*(**x**,*t*)—the instantaneous local uptake rate—is given by:

(5) $$ k(\mathbf{x}, t) = \rho(\mathbf{x}, t) \frac{E k_{\text{cat}}}{V_{\text{cell}}} \frac{\phi_{\text{ext}}(\mathbf{x}, t)}{k_{\text{m}} + \phi_{\text{ext}}(\mathbf{x}, t)}  $$

Michaelis-Menten kinetics has been applied in the past to the enzymatic uptake of substrates by cells (beginning as early as 1949 [25]). In the above expression, *ρ*(**x**,*t*) again represents the instantaneous local cell volume fraction, *E* represents the number of enzymatic transporters on a cell membrane, *V*_cell_ represents the volume of a cell, *k*_cat_ represents the enzyme turnover rate, and *k*_m_ is the so-called Michaelis constant for the reaction. In the case of glucose, the parameters $\frac {E k_{\text {cat}}}{m_{\text {cell}}}$ and *k*_m_ were fit to experimental measurements [16], yielding 10.40 mmol gDwt ^−1^ hr ^−1^ (a value in close agreement with a similar analysis in [26] and the default value in the *iJO1366* metabolic model) and 0.370 mM, respectively. Literature values for the average dry weight, *m*_cell_, and volume, *V*_cell_, of a single cell were then used to transform $\frac {E k_{\text {cat}}}{m_{\text {cell}}}$ to $\frac {E k_{\text {cat}}}{V_{\text {cell}}}$ (see Table 1).

#### Flux balance analysis kernel

FBA is used to model substrate utilization and production in the simulation. The maximum instantaneous specific (or mass-normalized) uptake rate, *v*_max_, of a given substrate by a cell in lattice site **x** during the time interval *Δτ* is assumed to be given as:

(6) $${} {\fontsize{8.2pt}{9.3pt}\selectfont{\begin{array}{ll} v_{\text{max}}(\mathbf{x}, t) \,=\, \left\{ \begin{array}{ll} \phi(\mathbf{x}, t) \frac{V_{\text{cell}}}{m_{\text{cell}} \Delta\tau} &\text{if \(\phi\) is passively transported into the cell}\\ \frac{\phi_{\text{int}}(\mathbf{x}, t)}{\rho(\mathbf{x}, t)} \frac{V_{\text{cell}}}{m_{\text{cell}} \Delta\tau} &\text{if \(\phi\) is actively transported into the cell} \end{array}\right. \end{array}}}  $$

This simply requires that during the time interval *Δτ* the cells have access only to the substrate lying within their volume— *ϕ*(**x**,*t*)*V*_cell_ for passively diffusing substrates (assuming the substrate is distributed uniformly throughout the lattice site), and $\frac {\phi _{\text {int}}(\mathbf {x}, t)}{\rho (\mathbf {x}, t)} V_{\text {cell}}$ for actively imported substrates (where the factor of *ρ*(**x**,*t*)^−1^ reflects the fact that *ϕ*_int_ is known to be confined entirely within the local cells). Constraints of this type are calculated for each metabolite that is tracked in the spatially-resolved simulation (see Figure 1C), and used with a genome-scale flux balance metabolic model in order to predict the behavior of the cells in each lattice site (see Figure 1D). The local substrate concentrations are then updated accordingly as:

(7) $$  \phi(\mathbf{x}, t + \Delta\tau) = \phi(\mathbf{x}, t) - v_{\text{FBA}}(\mathbf{x}, t) \frac{m_{\text{cell}} \Delta\tau}{V_{\text{cell}}} \rho(\mathbf{x}, t)  $$

where *v*_FBA_(**x**,*t*) represents the exchange flux for the substrate predicted by FBA. The second term on the right hand side can be thought of as the product of the predicted uptake rate per cell, *v*_FBA_(**x**,*t*)*m*_cell_, the number-density of cells in the lattice site, $\frac {\rho (\mathbf {x}, t)}{V_{\text {cell}}}$, and the time step.

Because simulations can easily involve on the order of 1 million cell-type lattice sites (ours involve approximately 1.6 × 10^6^), and because each site requires frequent updates to its substrate concentrations (ours are updated around 1,000 times per simulated second), a single second of typical simulation time can require on the order of 10^9^ or more individual FBA solutions. Many of these will be similar to each other; the behaviors of cells in adjacent lattice sites or in the same site in subsequent time steps will often not change appreciably, meaning that the same or nearly the same solution can be used over and over again, avoiding the need for redundant FBA solving. To that end we have approximated the local instantaneous FBA solution in a given lattice site using a precomputed lookup table (see Additional file 1: Section 1.2 for details).

#### Cell growth kernel and colony expansion kernel

The local instantaneous growth rates, *g*(**x**,*t*), predicted by FBA are used to update the volume fraction in each lattice site in accordance with an assumed exponential growth law:

(8) $$ \rho(\mathbf{x}, t + t_{\text{grow}}) = \rho(\mathbf{x}, t) e^{g(\mathbf{x}, t) t_{\text{grow}}}  $$

The volume fraction within a lattice site is allowed to increase until it surpasses *ρ*_max_. At this point the site is considered “over-filled,” and the force of the cells in the lattice site pushing against each other create an outward pressure through each of the lattice site’s six walls (see Figure 1E). Because the growth of the colony is slow, the cells are assumed to have ample time to redistribute themselves in responce to these intercellular forces, and as a result the cell volume fraction is assumed to remain relaxed throughout the colony at all times. This relaxation is performed immediately after application of the Growth kernel and involves the iterative movement of cell volume from over-filled sites to neighboring sites with lower volume fractions until the highest volume fraction in the entire colony is within some small user-defined cutoff, *Δρ*, of *ρ*_max_ (see Additional file 1: Figure S1). The amount of volume fraction, *ρ*_*i*→*j*_, moved from lattice site *i* to a neighboring site *j* in a single iteration is proportional to the difference in the sites’ respective degrees of over-filling:

(9) $$ \rho_{i \rightarrow j} = \frac{1}{12} [\text{max}(0, \rho_{i} - \rho_{\text{max}}) - \text{max}(0, \rho_{j} - \rho_{\text{max}})]  $$

Here, the factor of $\frac {1}{12} = \frac {1}{2} \times \frac {1}{6}$ accounts for the fact that each lattice site has six faces through which cell volume can be moved, and the factor of $\frac {1}{2}$ ensures convergence. In cases where *ρ*_*i*→*j*_<0, cell volume is moved from site *j* to site *i*. When multiple cell types are present in the same lattice site, the total volume fraction is used to determine how much cell material is moved across each face, and this is then divided up among the cell types according to their relative fractions.

For spherical cells, the maximum packing fraction, *ρ*_max_, might be set to approximately 0.74—the close-packing fraction of uniform spheres. The simulations presented here use the value 0.65 (accounting for approximately 650 cells per lattice site) in accordance with a model of the growth of colonies of rod-shaped *Salmonella typhimurium* [15].

#### Regulation kernel

Multiple different cell types can to be simulated simultaneously. These can be either different species or different regulatory states of the same species. In the latter case, the cells’ regulatory state is allowed to change over time in response to its environment (see Figure 1F). This is performed straightforwardly assuming first order kinetics:

(10) $${} {\fontsize{8.5pt}{9.3pt}\selectfont{\begin{aligned} \rho_{i}(\mathbf{x}, t + t_{\text{grow}}) &= \rho_{i}(\mathbf{x}, t) \,+\, \sum_{j} k_{j \rightarrow i}(\phi_{m}(\mathbf{x}, t), \phi_{n}(\mathbf{x}, t)) \rho_{j}(\mathbf{x}, t) t_{\text{grow}} \\ &\quad- k_{i \rightarrow j}(\phi_{o}(\mathbf{x}, t), \phi_{p}(\mathbf{x}, t)) \rho_{i}(\mathbf{x}, t) t_{\text{grow}} \end{aligned}}}  $$

where *ρ*_*i*_(**x**,*t*) represents the instantaneous local volume fraction of cell type *i* in lattice site **x**, and *k*_*j*→*i*_(*ϕ*_*m*_(**x**,*t*),*ϕ*_*n*_(**x**,*t*)) represents the instantaneous local switching rate from regulatory state *i* to *j*. These switching rates are assumed to depend on the local substrate concentrations (up to two of them— *ϕ*_*m*_ and *ϕ*_*n*_ or *ϕ*_*o*_ and *ϕ*_*p*_ above) and, for simplicity, be polynomial in form:

(11) $$ {\small{\begin{aligned} {} k_{i \rightarrow j}(\phi_{m}(\mathbf{x}, t), \phi_{n}(\mathbf{x}, t)) &= \text{max}(0, \alpha_{0} + \alpha_{1} \phi_{m}(\mathbf{x}, t) + \alpha_{2} \phi_{n}(\mathbf{x}, t) \\ &\quad + \alpha_{3} \phi_{m}(\mathbf{x}, t)^{2} + \alpha_{4} \phi_{n}(\mathbf{x}, t)^{2}\\ &\quad + \alpha_{5} \phi_{m}(\mathbf{x}, t) \phi_{n}(\mathbf{x}, t)) \end{aligned}}}  $$

where the constants {*α*_*k*_} are fit to experimental data or a known model. The simulations presented here include a “regulated” model (see below) that involves the switching of cells between glucose- and acetate-consuming states. The switching rate parameters for this model were fit to data from [27], (see Additional file 1: Figure S2).

### 3DdFBA predicts *E. coli* in colonies engage in cooperative acetate crossfeeding

#### Unregulated model

**Figure 2:**
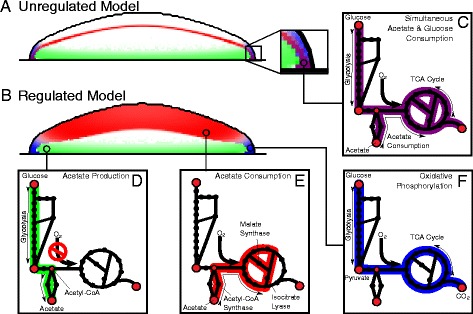
**Metabolic behaviors within the “unregulated” and “regulated” colony models.** **(A)** The unregulated model in cross-section after 32 hours of growth; cells were assumed to engage in their own optimal metabolism solely in response to the metabolites available. **(B)** The regulated model in cross-section after 32 hours of growth; cells were allowed to be in either a glucose-consuming or acetate-consuming state. **(C)** Cartoon of *E. coli* central metabolism. The purple color indicates flux through the metabolic network. Some cells of the unregulated model were predicted to engage in simultaneous glucose and acetate consumption; this highlights the necessity of accounting for resource regulation within the simulations. **(D)** Acetate production within both models occurred near the agar in the anoxic interior of the colony; there glucose was available but the lack of oxygen prevented use of the TCA cycle and electron transport chain. **(E)** Acetate consumption occurred as a thin dome within the unregulated model and as a wider and more diffuse dome in the regulated model. Also indicated are the Acetyl-CoA Synthase, Malate Synthase, and Isocitrate Lyase reaction steps. These are associated with acetate consumption and are catalyzed by Acs, AceB, and AceA (which is cotranscribed with with AceB in the *aceBAK* operon), respectively. **(F)** Oxidative phosphorylation occurred near the agar at the outer edge of the colony.

Initially, simulations were performed without any form of regulation. The cells were assumed to engage in the metabolism that optimized growth solely in response to the substrates available (see Figure 2A). A form of cooperative crossfeeding was found to emerge within the simulated colony wherein one subpopulation produced acetate while another consumed it. This behavior resulted predominantly from oxygen depletion in the colony interior. The penetration depth of oxygen (as measured near the agar surface) was calculated to be between 40 and 50 *μ*m—in strong agreement with previous experimental and theoretical values [13,28]. Beyond this depth, extreme hypoxia prohibited cells from making use of the tricarboxylic acid cycle (TCA) and electron transport chain, and as a result they engaged in a form of fermentative metabolism that produced acetate as a byproduct (see Figure 2A, green region). Because the availability of glucose fell dramatically with height above the agar, these cells formed a broad flat disk near the base of the colony. As the acetate diffused outward, some of it was taken up by aerobic cells nearer the periphery, which formed a thin shell of syntrophic acetate-consumers (see Figure 2A, red region). This shell was approximately 20 *μ*m thick and accounted for a colony-wide average acetate uptake rate of about 1.32 mmol gDwt ^−1^ hr ^−1^ at 36 hours of simulation time. This was nearly 85% of the colony’s average acetate production rate. Because crossfeeding among *E. coli* is generally associated with either multi-species consortium or long-term evolutionary experiments where genetically distinct crossfeeding sub-strains arise over many generations [29], its emergence within an isogenic colony on time scales as short as a few days is of particular interest.

#### Regulated model

However these “unregulated” simulations yielded some unrealistic behavior. Many of the cells predicted to be taking up acetate were also predicted to be taking up glucose at the same time (see Figure 2A, purple region). Experimentally, glucose and acetate consumption are known to be differentially regulated, and *E. coli* in batch culture generally exhaust almost all of the glucose available to them before switching over *en masse* to acetate metabolism [30]. In order to ensure that the crossfeeding observed was not merely an artifact of the inability of the model to account for this effect, a more refined model was constructed. Cells were allowed to be in either a glucose-consuming state (wherein an upper bound of 0.0 was imposed on the acetate uptake reaction flux) or an acetate-consuming state (wherein an upper bound of 0.0 was imposed on the glucose uptake reaction flux), and could interconvert between the two at rates that depended on the local glucose and acetate concentrations. These rates were fit to experimental batch-culture data [27] using a simple growth model:

(12) $$ \begin{aligned} {} \frac{dM_{\text{glc}}}{dt} &= g_{\text{glc}}M_{\text{glc}} + k_{\text{ace} \rightarrow \text{glc}}([glc], [ace])M_{\text{ace}}\\ &\quad- k_{\text{glc} \rightarrow \text{ace}}([glc], [ace])M_{\text{glc}}\\ {}\frac{dM_{\text{ace}}}{dt} &= g_{\text{ace}}M_{\text{ace}} + k_{\text{glc} \rightarrow \text{ace}}([glc], [ace])M_{\text{glc}}\\ &\quad- k_{\text{ace} \rightarrow \text{glc}}([glc], [ace])M_{\text{ace}} \\ {}\frac{d[glc]}{dt} &= -M_{\text{glc}} v_{\text{glc}} \\ {}\frac{d[ace]}{dt} &= M_{\text{glc}} \epsilon_{\text{ace}} - M_{\text{ace}} v_{\text{ace}} \end{aligned}  $$

where *M*_glc_ and *M*_ace_ represent the biomass of the glucose-consuming and acetate-consuming cells, respectively, *g*_glc_ and *g*_ace_ represent their growth rates, *v*_glc_ and *v*_ace_ represent the uptake rates of glucose and acetate, respectively, and *ε*_ace_ represents the acetate production rate by glucose-consumers. Because the experimental data includes growth curves for cultures growing in only glucose and only acetate, *g*_glc_ and *g*_ace_ were easily fit assuming an exponential form (see Additional file 1: Figure S2 A). Using these values and the glucose and acetate concentration curves from the same single substrate experiments, values for *v*_glc_, *v*_ace_, and *ε*_ace_ were fit (see Additional file 1: Figure S2 B magenta and blue, C blue). Finally, with these values fixed, the switching rate parameters, {*α*_*i*_}, that appear in Equation 11 were fit.

Overall, the modeled dynamics fit well with the experimental behavior, especially in the low-acetate regime where our spatially-resolved FBA simulations primarily exist. The glucose concentration curves show very good agreement (see Additional file 1: Figure S2 C), but at intermediate acetate concentrations the model overestimates acetate uptake (see Additional file 1: Figure S2 B green, cyan). We attribute this to the fact that real cells should experience some lag in switching from glucose-consuming to acetate-consuming behaviors. This lag is not represented in the model; the modeled cells switch out of the glucose-consuming state directly to acetate-consuming behavior. We expect that the addition of a third non-growing “retooling” state between these glycolytic and acetoclastic states might bring the model into better agreement. Nevertheless, because the highest acetate concentration recorded in our 3DdFBA simulations is approximately 5.8×10^−3^ M—laying well within the range of the blue curve in Additional file 1: Figure S2 B where the model best matches experiment—a more refined model would likely add significant computational complexity without offering much in the way of accuracy in return. The switching rate parameters ultimately used in the model are summarized in Additional file 1: Table S1.

Simulation of this “regulated” model again yielded acetate crossfeeding (see Figure 2B). As before, the acetate-producing subpopulation consisted of glucose-consuming cells located within the anoxic interior of the colony near the agar. The acetate-consumers again formed a shell, but it was wider and more diffuse than in the unregulated model. This shell extended all the way to the edge of the colony, and was comprised of a mixture of both acetate-consuming cells (up to approximately 10% by volume) and slow- or non-growing glucose-consumers. Little acetate-consumption occurred at the colony periphery near the agar surface; this was because the high glucose concentration in this region strongly suppressed the acetate-consuming state and instead drove the cells toward use of the TCA cycle and electron transport chain (see Figure 2B, blue region). In total, the acetate-consumers accounted for a colony-wide average uptake rate of approximately 0.69 mmol gDwt ^−1^ hr ^−1^ at 36 hours of simulation time. This was only about 39% of the colony’s acetate production rate.

The ability to crossfeed acetate imparted a fitness advantage to the colony as a whole, and after 36 hours the model with regulation had outgrown a non-crossfeeding model (that was unable to consume acetate) by about 4.5%. This faster growth derived from the crossfeeding colony’s cells’ collective ability to aerobically metabolize glucose even when they would not have been able to individually. The acetate-producers lacked the oxygen necessary to fully metabolize glucose, and as a result could only partially metabolized it to acetate. The acetate-consumers higher up in the colony, which had access to oxygen but not glucose, were then able to complete the process by metabolizing the acetate. The cells of the non-crossfeeding model could not complete the second part of this two-step metabolism, and grew slower as a result.

Because high glucose availability strongly suppresses the acetate-consuming state, it was initially unclear if the crossfeeding observed could be disrupted by increasing the concentration of glucose in the agar. Additional simulations were performed with glucose concentrations spanning from 1.25 g l ^−1^ up through 10 g l ^−1^. In each case acetate crossfeeding emerged within the simulated colony (see Additional file 1: Figure S3) indicating that this behavior is fairly robust across a range of glucose concentrations.

#### 3DdFBA with molecular crowding

A cell’s finite volume places an inherent upper limit on the total number of metabolic enzymes that the cell can contain. Because every enzyme takes up some portion of this volume, and because the maximum flux through a given enzyme-mediated reaction is proportional to the number of enzymes that are present inside the cell, increasing the flux through a given metabolic pathway results in an increase in demand for the cell’s available space. Flux balance analysis with molecular crowding (FBAwMC) was developed to account for this effect, and has been shown to reproduce the bacterial Warburg effect in fast-growing *E. coli* populations, as well as the preferential utilization of some carbon sources over others (e.g. glucose over acetate) [31-34]. Additional 3DdFBA simulations—both with and without regulation—were performed using lookup tables generated with the FBAwMC approach (see Additional file 1: section 1.3 for methodological details).

The addition of crowding constraints did not disrupt the simulated colonies’ ability to crossfeed acetate. In fact, fairly little changed between the simulations using the standard FBA formulation and those accounting for molecular crowding. The greatest qualitative difference occurred among the fast-growing aerobic cells of the colony periphery near the agar surface. Without regulation, standard FBA speciously predicted that these cells would engage in simultaneous glucose and acetate utilization (which served as part of the impetus for the development of the regulated model). In the FBAwMC formulation, no such simultaneous glucose and acetate utilization was observed (see Additional file 1: Figure S4 A). This is because acetate, which has a lower metabolic yield than glucose, requires a comparatively larger total enzyme-mediated reaction flux in order to produce the same amount of biomass. This in turn means that an increase in growth rate due to acetate utilization costs more in terms of enzyme volume than the same increase in growth rate due to glucose utilization. Fast-growing cells, which are at or near their crowding limit, are therefore driven to utilize glucose exclusively, and only begin to scavenge acetate when glucose availability drops.

Crowding constraints also drove a small subset of the same fast-growing peripheral cells toward acetate production, even in the presence of ample oxygen. Without crowding constraints, these cells had engaged in rapid glycolytic growth that made heavy use of the cells’ oxidative phosphorylation machinery. This led to doubling times of around 41 minutes for the fastest-growing cells and no appreciable acetate production. When crowding constraints were introduced, the volumetric cost associated with the enzymes of the TCA cycle and electron transport chain drove the fastest-growing of these cells toward a mixed strategy of partial oxidative phosphorylation and partial overflow metabolism (wherein glucose was metabolized to acetate and excreted, see Additional file 1: Figure S4 C). This led to slower growth rates (doubling times increased to nearly 50 minutes for the fastest-growing cells) and significant acetate generation (∼3.5 mmol gDwt ^−1^ hr ^−1^, or roughly 33% of the maximal production rate among the anaerobic cells of the colony interior).

### Experimental support for the predicted crossfeeding behavior

**Figure 3:**
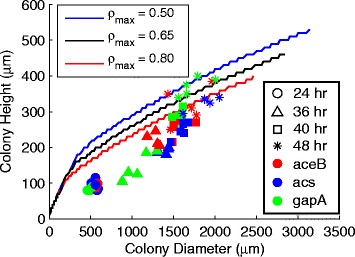
**Plot of simulated and experimental colony heights and diameters.** Plot of simulated and experimental colony heights and diameters. At 24 hours (circles), 36 hours (triangles), 40 hours (squares), and 48 hours (stars) after inoculation onto agar plates, 5 colonies of each of our fluorescent strains, PaceB-*gfp* (red), Pacs-*gfp* (blue), and PgapA-*gfp* (green), were imaged and measured. The lines indicate the height and width of modeled colonies (with regulation) over 48 hours of growth. These colonies were simulated with different values for *ρ*_max_ ranging from 0.50 to 0.80. The main simulations presented in the text use a value of 0.65 taken from the literature [15], and appear as the black line. The step-like features along these lines are artifacts of the discreteness of the spatial model. The simulations overestimate colony height early on, but their height-to-width ratios shows strong agreement at later time points.

A simple set of experiments was devised in order to test for the predicted crossfeeding behavior. *E. coli* K-12 strains containing a plasmid expressing GFP under the control of the malate synthase A (*aceB*) promoter were grown on agar plates containing M9 salts supplemented with 2.5 g l ^−1^ glucose and trace elements. This gene is part of the acetate operon, *aceBAK*, and is associated with acetate consumption (see Figure 2E). Colonies were grown over a period of two days during which time they were periodically (24, 36, 40, and 48 hours after plating) imaged at a series of focal planes above the agar surface. Imaging was performed using a Zeiss Axio Zoom.V16 microscope equipped with a Zeiss ApoTome.2 structured illumination device for optical sectioning [35]. The resulting heights, widths, and spatial distributions of fluorescence—indicative of the distribution of acetate-utilizing cells within the colonies—were then compared against those of the simulated colonies (see Figure 3, and Additional file 1: Figure S5).

**Figure 4:**
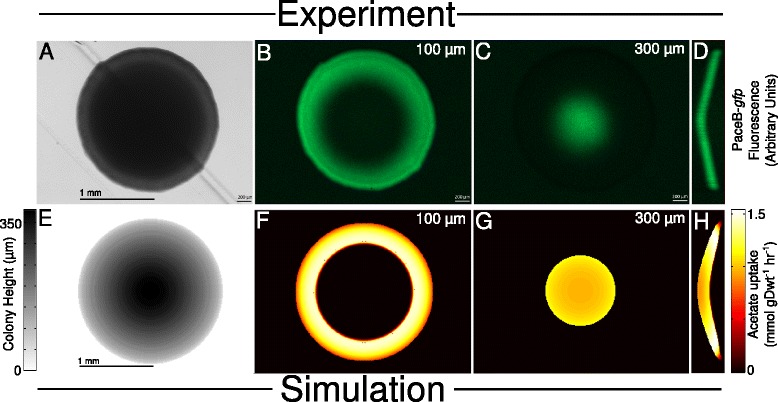
**Comparison of experimental acetate-associated reporter expression with predicted acetate consumption.** **(A)** Brightfield image of a representative colony expressing GFP under the colntrol of the *aceB* promoter. This image was taken approximately 48 hours after innoculation when the colony was approximately 2.0 mm in diameter. **(B)** PaceB-*gfp* fluorescence in the same colony imaged 100 *μ*m above and parallel to the agar surface. **(C)** PaceB-*gfp* fluorescence imaged 300 *μ*m above the agar surface. **(D)** PaceB-*gfp* fluorescence in a plane bisecting the colony and perpendicular to the agar surface; this was reconstructed from the entire compiled Z-stack of fluorescence images. **(E)** Gray-scale plot of the height of a simulated colony. This image was produced after approximately 40 hours of simulation time when the colony was around 2.0 mm in diameter. **(F)** Predicted acetate uptake rate imaged 100 *μ*m above and parallel to the agar surface. **(G)** Predicted acetate uptake rate imaged 300 *μ*m above the agar surface. **(H)** Predicted acetate uptake rate in A plane bisecting the simulated colony and perpendicular to the agar surface.

Within the experimental colonies, rings of fluorescence— indicating the presence of cells on the colonies’ peripheries rapidly expressing the *aceB* gene—were observed. These rings narrowed at higher focal planes and eventually closed to a spot, indicating the height of the colony (which, for example, at the 48 hour time point ranged from approximately 250 to 400 *μ*m). Compiled together as a Z-stack, these rings form domes of fluorescence on the colonies’ peripheries that show strong qualitative agreement with the simulated results (see Figure 4A–H). Comparison with reference colonies expressing GFP from the promoter of the highly-expressed housekeeping gene *gapA* (see Additional file 1: Section 1.4.1 and Additional file 1: Figure S6) indicated that *aceB* expression was over seven-fold higher in our experimental colonies than in published results for cells grown in liquid culture [36] (see Additional file 1: Table S2 for details). Similar spatial patterns were also obtained using strains expressing GFP under the control of the acetyl-CoA synthase (*acs*) promoter which, like *aceB*, is associated with acetate consumption (see Additional file 1: Figure S7). In this case the ratio of *acs*-associated to *gapA*-associated GFP fluorescence was approximately 50-fold higher than published values for liquid-cultured cells.

**Figure 5:**
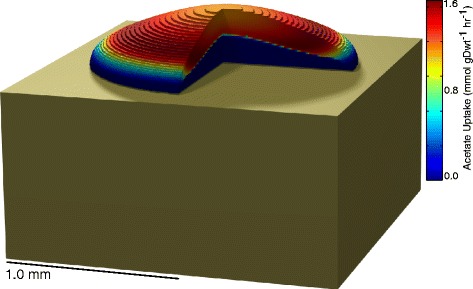
**Modeled three-dimensional** ***E. coli*** **colony.** This colony, approximately 32 hours after innoculation on an agar plate (tan region), is colored by acetate uptake rate.

Estimating the colony *acs* expression based on the fluorescence ratio computed above and published *gapA* expression data [37] yields an average value of approximately 704 Acs proteins per cell, most of which should be concentrated at the top and sides of the colony (Figures S6 & S7). The product of this and a published value for the Acs turnover rate (from *Salmonella enterica*, the only gram-negative bacterium with a wild-type Acs turnover rate listed in the Brenda database [38,39]) yields a maximum acetyl-CoA synthase reaction flux of 1.64 mmol gDwt ^−1^ hr ^−1^, in strong agreement with the maximum value of 1.66 mmol gDwt ^−1^ hr ^−1^ (or 1.60 mmol gDwt ^−1^ hr ^−1^ with crowding constraints imposed) predicted by the regulated models (see Figure 5). The unregulated models, by contrast, overestimated this value by approximately an order of magnitude. A similar calculation using the *aceB* fluorescence ratio and a published value for the AceB turnover rate [39,40] yields values of approximately 1,111 proteins per cell and a maximum flux of 1.24 mmol gDwt ^−1^ hr ^−1^, also in agreement with the regulated models.

Taken together, these experiments largely support the simulated results. Not only do the observed *aceB* and *acs* to *gapA* ratios indicate upregulation of genes associated with the predicted crossfeeding behavior, but this upregulation also occurs in spatial patterns similar to those seen in the simulations.

Additional control experiments are described in the Additional file 1: Sections 1.4.2–1.4.5. The first of these shows that the lack of fluorescence seen in the colonys’ interiors is not an artifact of the low oxygen concentration in these regions preventing GFP maturation. Colonies of *E. coli* expressing the flavin-based fluorophore iLOV [41,42]—which does not require oxygen to mature—show similar fluorescence patterns (see Additional file 1: Figure S8), meaning that the GFP fluorescence we see is likely indicative of gene-expression and not oxygen availability. The second control experiment addressed whether scattering of the excitation or emission photons as they passed through the colony might have obscured fluorescence in the interior. Fluorescent colonies were physically bisected and imaged at their cut plane (see Additional file 1: Figure S9). The resulting images show the same dome-like distribution of fluorescence seen using the non-disruptive structured illumination imaging technique (see Additional file 1: Figure S8), indicating that this distribution is not simply an artifact of the imaging technique used. A third control experiment involved colonies containing the promoterless plasmid pUA66—the same plasmid used to study *aceB*, *acs*, and *gapA* except without a promoter region upstream of the *gfp* gene. No appreciable fluorescence was seen, indicating that the fluorescence of our experimental colonies is not simply misattributed autofluorescence or leaky transcription of the plasmid *gfp* (see Additional file 1: Figure S11). Finally, we performed a preliminary two-color experiment using a plasmid containing the gene encoding mCherry under the control of the *ptsG* promoter (part of the glucose phosphotranspherase system) and *gfp* under the control of the *aceB* promoter (see Additional file 1: Figure S12). Although we note that the growth of the resulting colonies was slow and the GFP fluorescence we observed was noticeably less intense than that of our single-color experiments, we found that the resulting images strongly indicate the existence of distinct glucose- and acetate-consuming subpopulations.

### 3DdFBA predicts realistic colony growth dynamics

**Figure 6:**
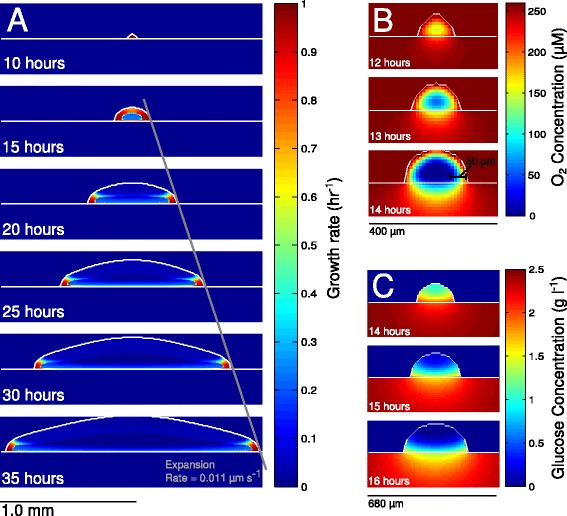
**Growth rates and substrate profiles over time.** **(A)** The colony is colored by growth rate and shown in cross-section. The fastest-growing cells (red) inhabit the colony periphery, while much of the interior shows little or no growth (blue) due to nutrient depletion. The grey diagonal line shows the linear radial growth of the colony. **(B)** Oxygen concentration within the same colony in cross-section at 12, 13, and 14 hours. Between 13 and 14 hours, a well-defined anoxic region forms in the center of the colony. The penetration of oxygen into this colony is between 50 and 60 *μ*m. **(C)** Glucose concentration in cross-section at 14, 15, and 16 hours. Beyond 14 hours, the glucose concentration in the colony interior rapidly falls, and beyond 15 hours, much of the colony interior, in addition to being anoxic, is also glucose-starved.

The physical growth of the simulated colonies was found to proceed through two phases. During the initial 15 hours, the dimensions of the colonies grew approximately exponentially. Beyond this time, however, the colonies’ radial expansions slowed to a constant rate of about 0.011 *μ*m s ^−1^ (see Figure 6A). These findings agree extremely well with an experimental study of *E. coli* growth under nearly identical conditions (solid agar medium with M9 salts, glucose, and trace elements) that reported an exponential-to-linear transition occurring around 12 hours after inoculation onto agar plates and a radial expansion rate of around 0.008 *μ*m s ^−1^ [28]. Our own experimental colonies (on the same solid medium) grew slightly slower with a radial expansion rate of approximately 0.007 *μ*m s ^−1^ (see Additional file 1: Figure S5).

The observed shift toward linear expansion was, like the predicted acetate crossfeeding, the result of nutrient depletion in the interior of the colony, and both oxygen and glucose starvation were found to contribute. After approximately 13 hours of simulation time, the colony had grown large enough to permit the emergence of clearly defined aerobic and anaerobic regions (see Figure 6B). Roughly commensurate with this drop in oxygen availability in the colony interior came a drop in glucose availability (see Figure 6C). The concentration of diffusing glucose at the center of the colony (as measured at its radial center and half its instantaneous height) fell to approximately half its initial value within 14 hours and about 4% of its initial value within 16 hours. Combined, these oxygen and glucose gradients gave rise to a relatively compact ring of fast-growing “pioneer” cells at the colony edge and almost no growth among the cells of the colony center, in agreement with experimental observations [43] (see Figure 6A). Because this ring’s height, width, and growth rate were controlled by the penetration depths of oxygen and glucose, they remained relatively constant over most of the latter part of the simulations. For this reason, the rate at which the biomass of the colony increased was proportional to its radius alone, and in turn, its radial expansion rate remained approximately constant (see Additional file 1: Section 1.5 for details).

The transition from exponential to linear growth also affected the shape of the colony. Early on, when oxygen and glucose were essentially ubiquitous, the simulated colony grew hemispherically in shape. Later, as the majority of the growth shifted to the periphery, the colony’s radial expansion outpaced its vertical growth, and it took on a more broad and flat appearance. Experimentally, the early growth of a colony is predominantly two dimensional across the agar [44,45], meaning that our simulations significantly overestimate the height of small colonies. Despite this initial divergence, later time points show better agreement with the height-to-diameter ratios of the experimental colonies (see Figure 3, black line). Varying the maximum local volume fraction, *ρ*_max_, toward higher values brings the simulations into even better agreement (see Figure 3 and Additional file 1: Figure S5, red lines). It is worth noting that our model does not require any sort of height parameterization; the agreement we see emerges naturally from the way the colony growth and expansion is handled in the simulation.

## Conclusions

The application of the 3DdFBA method described here focused on the growth and collective metabolism of *E. coli* colonies. Its integration of 3D reaction-diffusion simulations with a flux balance model of metabolism that involves thousands of reactions and metabolites enabled us to generate new hypotheses that we then tested directly in the laboratory.

The most striking result of this work was the prediction that subpopulations within *E. coli* colonies naturally engage in cooperative acetate crossfeeding. This was not due to the evolution of distinct crossfeeding genotypes, as is known to occur in long-term continuous culture experiments, but rather it emerged from the cells’ own regulatory responses to their local microenvironments within the colony. Depending on location, some cells experienced a glucose-rich anoxic environment that drove them toward acetate-producing fermentative metabolism, while others experienced a glucose-poor aerobic environment that favored acetate-consumption. This behavior remained robust over a range of common agar glucose concentrations, meaning that it may be occurring—completely unnoticed—in laboratory incubators the world over.

The simulated colonies exhibited realistic growth dynamics. The same glucose and oxygen gradients that gave rise to acetate crossfeeding also gave rise to a ring of fast-growing pioneer cells at the colony’s edge, and significantly hindered the growth of much of the colony interior. The pioneer ring had a profound impact on the macroscopic shape and growth of the simulated colony, leading to both its broad and flat appearance and its linear radial expansion (both of which agree well with experimental values).

Several features of our modeling technique proved essential to our study. The first, and most critical, was its ability to perform 3D simulations with fine spatial resolution. The concentration profiles of oxygen and glucose within the colony changed dramatically over short distances in both the radial and vertical directions. The penetration depth of oxygen, for example, was only around 50 *μ*m from the edge of the colony, while the penetration depth of glucose at the colony center was only around 60 *μ*m upward from the agar. Accurately accounting for these steep gradients required the use of a 3D lattice with a spacing of on the order of 10 *μ*m. Although a similar method was recently reported [21], it was restricted to two spatial dimensions and its reliance on costly on-the-fly FBA calculations severely limit its practicality for performing the millions of metabolism evaluations per-timestep necessary to resolve these profiles. In contrast, our use of precomputed FBA lookup tables enabled us to preform our simulations at nearly real-time speeds. Our code, running on the Keeneland supercomputer (with Nvidia M2090 GPUs) and on a Linux desktop machine (with a single Nvidia GTX780 GPU) performed the simulations presented here at speeds of approximately 50.0 and 40.1 simulated minutes per wall-clock hour, respectively. The second feature that proved critical was our method’s ability to account for the regulation of resource use by the modeled cells. Naively using FBA alone led to some cells simultaneously taking up glucose and acetate in a manner at odds with experimental data. By requiring the colony to obey a phenomenological model of the acetate switch, this pathological behavior was ameliorated, and the potential for acetate crossfeeding to emerge within wild-type *E. coli* colonies was more realistically modeled.

It is worth noting that our use of precomputed FBA tables, while offering vast speedup over on-the-fly evaluation, does carry some drawbacks. The most pressing of these is that the modeler must have some notion of the substrates that are likely to play a role in the colony’s collective metabolism before a simulation can be launched. Our choice to model only glucose, oxygen, and acetate was informed by earlier work simulating significantly smaller clusters of cells [10] and some preliminary FBA calculations. Nevertheless, this choice can in some ways limit the scope of the simulations. For example we knowingly ignore the potential for succinate crossfeeding (which was deemed unimportant due to the low predicted succinate production rate of anaerobically-growing cells) as well as possible toxic effects due to the production of ethanol within the colony (although, because the ethanol and acetate production rates are comparable, the concentration of ethanol should not rise significantly above the maximal acetate concentration, ∼5.8×10^−3^ M, which is approximately 2 orders of magnitude below the concentration at which cellular growth is significantly inhibited [46]). Incorporating larger numbers of substrates into a lookup table is straightforward, but can be time consuming as it increases the dimensionality of the table, and in turn, the number of FBA calculations required to produce it. Ultimately, the choice of using table lookups or on-the-fly FBA solving comes down to a choice between computational speed/resolution and model flexibility/universality. With enough prior knowledge of the phenomenon to be simulated, a table-based method vastly out-performs on-the-fly solving, but for purely exploratory simulations in which large numbers of metabolites are simulated simultaneously, an on-the-fly method may potentially yield novel behaviors that the modeler did not anticipate.

Although our methodology represents an important step in using FBA in both a spatially- and temporally-resolved manner, neither it, nor any other current implementation, can fully account for all biologically significant phenomena. One important example stems from the inherent determinism of the method which yields only average behaviors. Stochastic gene expression has been shown to give rise to significant metabolic variability, even among cells in otherwise identical environments [6]. The method we present does not account for this type of cell-to-cell variability; the cells in a given lattice site are assumed to behave identically, engaging in the optimal metabolism possible given their local substrate availability. Additional uncertainties arise in the number of cells that actually seed a given colony; our simulations assume a single cell, but it is difficult to experimentally verify the exact number of cells left in a given location when an agar plate is streaked. These effects are evident even at the macroscopic level—while the heights and widths of experimental colonies show considerable variability (see Figure 6), our simulated colonies show none. A natural way forward in this regard is to shift toward an agent-based modeling approach where individual cells may be represented within a continuous field of diffusing substrates. This could allow for stochastic gene expression to be explicitly accounted for within each cell; the gene expression state of a cell might be sampled from experimental distributions and used to apply constraints within the cell’s metabolic network in a manner similar to that of [6]. Additionally, by resolving individual cells and their intercellular forces, an agent-based approach may better account for the early development of the colony as it transitions from 2D to 3D growth [44]—a behavior that is poorly accounted for in our current implementation.

Our modeling technique can be applied to the study of communities involving many different microbial species. Several different cell types—each employing their own metabolic model—can be simultaneously simulated, and because the different cell types can transform into each other, regulation systems much more complex than the acetate switch described here can be studied. Beyond obvious future studies of biofilms, perhaps the most exciting applications of this technique may come in the form of tissue and tumor modeling. Like the colony models presented here, steep oxygen gradients are known to form within cancerous tumors that profoundly affect their metabolism [47]. Within certain cancers these oxygen gradients have even been reported to give rise to lactate crossfeeding in a manner strikingly similar to the acetate crossfeeding seen in our simulations [48,49]. There are already several flux balance models of different human cancers available in the literature [50,51]. These can be leveraged to build new 3D models capable of studying everything from environmental fluctuations and metabolic reprogramming within a cancer to the interactions between cancers and their surrounding tissues.

## Methods

### Simulation methods

All FBA calculations were performed using the freely available COBRA toolbox [3] and the iJO1366 E. coli genome-scale metabolic model [4,52] with default uptake rates for M9 salts and trace elements. FBAwMC calculations were performed using crowding coefficients taken from [31]. Our 3DdFBA simulation code outlined in Algorithm 1 was written in CUDA and C/C++ and was run on the Keeneland GPU supercomputer (NVIDIA M2090 GPUs), our own GPU cluster (NVIDIA C2050 GPUs), as well as a desktop workstation with a single NVIDIA GTX780 GPU. The simulations required approximately 2.2 GiB memory and achieved speeds of approximately 27.5 simulated minutes per wall-clock hour on the C2050s, 40.1 simulated minutes per wall-clock hour on the GTX780, and 50.0 simulated minutes per wall clock hour on the M2090s. Our code is available at http://www.scs.illinois.edu/schulten/software/index.html.

### Microbiological methods

The E. coli strain and plasmids containing gfp under the control of the aceB, the acs, and the gapA promoters that were used in this study are listed in Additional file 1: Table S3. Also listed are a promoterless plasmid used as a negative control (see Additional file 1: Section 1.4.4 and Additional file 1: Figure S11), and a plasmid containing the iLOV [41] gene under control of the constitutive phage *λ* promoter which was used as another control. The bacteria were grown at 37 °C in liquid culture (LB) and on solid medium (1.5% agar with 2.5 g l ^−1^ glucose, M9 salts, and trace elements) containing 25 *μ*g/ml kanamycin (Km) [53]. The trace element solution (including FeSO _4_, ZnSO _4_, MnSO _4_, CuSO _4_, CoCl _2_, sodium borate, sodium molydbate, and ethylenediaminetetra-acetic acid (EDTA)) used was prepared in accordance with [28]. Liquid cultures were grown in a shaking incubator.

### Construction of a transcriptional gapA promoter-gfp fusion

The preparation of plasmid DNA, DNA digests, agarose gel electrophoresis, cloning, and transformation of E. coli cells were performed following established protocols [53]. The DNA fragment containing the promoter region of gapA was PCR amplified from a liquid E. coli K12 culture with primers that were engineered to contain a XhoI 5’ end and a BamHI 3’ end (see Additional file 1: Table S4). The PCR products were cloned into the low copy number cloning vector pUA66 (Additional file 1: Table S3), bringing the PCR products in correct orientation to exert control over gfp nexpression. The resulting plasmid carrying the gapA promoter fusion was introduced into E. coli K12 via electroporation. The correct promoter insert was confirmed for the plasmids via PCR.

### Imaging of bacterial colonies

Bacterial colonies for imaging were grown on M9 medium agar plates supplemented with 0.25% glucose, trace elements, and Km. Imaging was performed at 24, 36, 40, and 48 hours after innoculation. Bright field and fluorescence images were captured using an Axio Zoom.V16 fluorescence microscope equipped with an ApoTome.2 structured illumination device (Zeiss) for optical sectioning. This microscope was chosen because it enabled observation of individual colonies growing directly on streaked plates without requiring the preparation of specialized agar-coated microscope slides or any physical disruption of the colonies or agar. Five individual colonies were viewed during 2 independent experiments. Each colony was imaged from bottom to top by optical sectioning in 5 *μ*m steps. The Zen 2011 software (Zeiss) was used to create images and the AxioVision (Zeiss) software was used for calculating the average fluorescence intensity of each of the 40-hour colonies (chosen because these colonies were large–roughly 1.5 mm in diameter—but still significantly smaller than the 3.2 mm agar pad used in our simulations, thus avoiding possible boundary effects when comparing simulation and experiment). This average was computed over the cylinder whose base inscribes the bottom of the colony and whose height is the same as the colony; at each imaged plane, the average intensity within the circular projection of the base onto the plane was computed, then all of these values were averaged to give the average over the entire cylinder. Colony diameters were measured using the bright field images and colony heights were measured as the distance between the first and last focal planes that clearly indicated fluorescence. In addition to the 40 hour time point, heights and diameters were computed for colonies grown for 24, 36 and 48 hours. Finally, cells grown in liquid culture were imaged using a Zeiss Axiovert 200M microscope. These images showed that the presence of the various plasmids used in our study did not effect the architecture of the cells (see Additional file 1: Figure S13).

## Additional file

Additional file 1
**Supporting Information Text, Tables, and Figures.** PDF document containing supporting information, including text, tables and figures.

## Abbreviations

FBA: Flux balance analysis
3DdFBA: Three-dimensional dynamic flux balance analysis
FBAwMC: Flux balance analysis with molecular crowding
TCA: Tricarboxylic acid
