# Supplementary material for: Spatially-resolved metabolic cooperativity within dense bacterial colonies
Source: BMC Syst Biol. 2015 Mar 18;9:15. doi: 10.1186/s12918-015-0155-1 (PMC4376365; doi:10.1186/s12918-015-0155-1)
Supplement: Additional file 1 — Supporting Information Text, Tables, and Figures. PDF document containing supporting information, including text, tables and figures. [file 12918_2015_155_MOESM1_ESM.pdf]

Additional file 1: Supporting Information Text, Tables, and Figures
Spatially-Resolved Metabolic Cooperativity Within Dense Bacterial Colonies

John A. Cole, Lars Kohler, Jamila Hedhli, and Zaida Luthey-Schulten

March 3, 2015

Contents

1 Additional file 1: Supporting Information Text 1

1.1 Additional file 1: Choosing t_{ss} and t_{grow} 1

1.2 Additional file 1: FBA Table Lookup 2

1.3 Additional file 1: FBA with Molecular Crowding 3

1.4 Additional file 1: Experimental Controls 3

1.4.1 Additional file 1: PgapA-*gfp* Expression 3

1.4.2 Additional file 1: Anaerobic Fluorophores 4

1.4.3 Additional file 1: Physical Cross-sections 4

1.4.4 Additional file 1: Promoterless GFP Expression 5

1.4.5 Additional file 1: Preliminary Two-Color Experiment 5

1.5 Additional file 1: Linear Radial Growth of Colonies 6

2 Additional file 1: Supporting Information Tables 6

3 Additional file 1: Supporting Information Figures 9

1 Additional file 1: Supporting Information Text

1.1 Additional file 1: Choosing t_{ss} and t_{grow}

The value for t_{ss} was chosen based on the lattice spacing, λ, and the diffusion rate of the slowest diffusing chemical species, D_{slow} in the simulation. At every t_{grow}, colony growth and expansion computations are performed which expand the front of the colony. This growing front adds new space for the metabolites to diffuse into. Ideally, this new space should not be wider than a single

lattice spacing (which sets an upper limit on the length of t_{grow}, see below). The expected time required for the slowest diffusing metabolite to diffuse throughout a shell of width λ then sets a lower limit for the chemical equilibration time of $t_{\text{ss}} \gg \frac{\lambda^2}{D_{\text{slow}}}$. In our simulations, $\lambda = 10$ μm, and glucose is the slowest diffusing metabolite with $D_{\text{glc}} = 7.8 \times 10^{-10}$ m^2 s^{-1} [1], leading to a requirement of $t_{\text{eq}} \gg 0.13$ s. A value of $t_{\text{eq}} = 1$ s was chosen for our simulations.

As mentioned above, an upper limit for t_{grow} can be estimated based on the lattice spacing and the expected radial growth rate, $\dot{r}$, of the modeled colony. Literature values for $\dot{r}$ range up to approximately 0.013 μm s^{-1} [2], which means that ensuring that the colony front expands significantly less than a single lattice spacing during t_{grow} requires $t_{\text{grow}} \ll \frac{\lambda}{\dot{r}} \sim 770s$. A conservative value of 60 s was chosen for our simulations.

1.2 Additional file 1: FBA Table Lookup

In order to avoid costly on-the-fly FBA computations, an FBA lookup table is generated prior to simulation launch by systematically stepping through the possible uptake rate upper bounds for each of the modeled substrates, and performing parsimonious FBA (or pFBA) [3] at each step. The step size, δ_i, for the ith substrate, is chosen to be small; in the case of the simulations presented here, the glucose, oxygen, and acetate uptake rate upper bounds were stepped from values of 0.0 to 10.4, 31.8, and 16.0 mmol gDwt^{-1} hr^{-1}, respectively (see Table 1 in the main manuscript for references), in steps of size 0.2 mmol gDwt^{-1} hr^{-1}. The resulting predicted uptake, efflux, and growth rates are then stored in a formatted text file that is read by the simulation program. All FBA calculations were performed using the freely available COBRA toolbox 2.0 [4].

Given maximum uptake rates $v_0, v_1 \dots v_n$ (computed according to Equation 6 of the main text) for the n substrates being modeled, the approximate flux for the ith substrate predicted by FBA is given by:

$$v_{\text{FBA},i} \approx T_i(\lfloor \frac{v_0}{\delta_0} \rfloor \delta_0, \dots, \lfloor \frac{v_n}{\delta_n} \rfloor \delta_n) + \sum_j \frac{\Delta_j T_i(\lfloor \frac{v_0}{\delta_0} \rfloor \delta_0, \dots, \lfloor \frac{v_n}{\delta_n} \rfloor \delta_n)}{\delta_j} \times \Delta v_j \tag{1}$$

In this equation $T(\lfloor \frac{v_0}{\delta_0} \rfloor \delta_0, \dots, \lfloor \frac{v_n}{\delta_n} \rfloor \delta_n)$, represents the FBA solution (the predicted optimal set of uptake and efflux fluxes and the growth rate) from the precomputed table generated using the set of uptake rate upper bounds $\{\lfloor \frac{v_0}{\delta_0} \rfloor \delta_0, \dots, \lfloor \frac{v_n}{\delta_n} \rfloor \delta_n\}$, and the subscript i denotes the ith substrate's flux from this solution. It is important to note that because the the table is generated by stepping through a range of flux upper bounds in increments of δ_i, the arguments of T must be rounded (down for simplicity) to integer multiples of δ_i. The second term represents a correction to this tabled solution; it is the sum of the residuals, $\Delta v_j = v_j - \lfloor \frac{v_j}{\delta_j} \rfloor \delta_j$, multiplied by $\frac{\Delta_j T_i(\lfloor \frac{v_0}{\delta_0} \rfloor \delta_0, \dots, \lfloor \frac{v_n}{\delta_n} \rfloor \delta_n)}{\delta_j}$, the approximate change in the ith substrate's predicted flux with respect to a change in the jth substrate's uptake rate upper bound. This is computed from the tabled solutions using:

$$\begin{aligned} \Delta_j T_i(\lfloor \frac{v_0}{\delta_0} \rfloor \delta_0, \dots, \lfloor \frac{v_n}{\delta_n} \rfloor \delta_n) = {} & T_i(\lfloor \frac{v_0}{\delta_0} \rfloor \delta_0, \dots, \lceil \frac{v_j}{\delta_j} \rceil \delta_j, \dots, \lfloor \frac{v_n}{\delta_n} \rfloor \delta_n) \\ & - T_i(\lfloor \frac{v_0}{\delta_0} \rfloor \delta_0, \dots, \lfloor \frac{v_j}{\delta_j} \rfloor \delta_j, \dots, \lfloor \frac{v_n}{\delta_n} \rfloor \delta_n) \end{aligned} \tag{2}$$

where it should be noted that the jth argument in the first term on the RHS is computed using the ceiling function ($\lceil\rceil$), while in the second term it is computed using the floor function ($\lfloor\rfloor$). These approximate v_{FBA} values are then used to update the local substrate concentrations in accordance with Equation 7 of the main text.

1.3 Additional file 1: FBA with Molecular Crowding

The FBA with Molecular Crowding (FBAwMC) methodology was developed in [5]. Briefly, for a cell of volume V, the number, n_i, of enzymes of type i within the cell is constrained by:

$$\sum_{i=1}^{N} v_i n_i \leq V \quad (3)$$

where v_i is the volume of the enzyme. Assuming a fixed cytoplasmic density, $C \approx 0.34$ g ml^{-1}, and that the maximum flux through a given reaction can be written as the product of the copy number of the enzyme that catalyzes it and the enzyme's turnover rate, k_{cat}, Equation 3 can be rewritten as:

$$\sum_{i=1}^{N} a_i f_i \leq 1 \quad (4)$$

where $a_i = C v_i / k_{\text{cat}}$ is the "crowding coefficient" and f_i is the flux through reaction i.

Estimates of a_i for 109 reactions were reported in [5]. These values spanned several orders of magnitude and contained a number of outliers. The 15 lowest and highest values were removed, and of the remaining 79, the 64 that corresponded to reactions in the *iJO1366* metabolic reconstruction were used to impose crowding constraints. The rest of the reactions were constrained using estimates of a_i that were based on the sequence length, l_i, of the enzyme that catalyzes each reaction. Assuming that $v_i \propto l_i \Rightarrow a_i \propto l_i$, an approximate proportionality constant was found between the central 64 values of a_i mentioned above and the sequence lengths of their respective enzymes (sequence lengths were taken from the *E. coli* K12 MG1655 uid 57779 genome available at ftp://ftp.ncbi.nlm.nih.gov/genomes/Bacteria/Escherichia_coli_K_12_substr__MG1655_uid57779/). This proportionality was found to be $\sim 4.67 \times 10^{-6}$ hr gDwt mmol^{-1} per amino acid. The pearson correlation coefficient between sequence length and a_i was found to be 0.51. This led to an average crowding coefficient over all reactions of $\langle a \rangle \approx 0.002$, which is roughly half of that reported in [5].

1.4 Additional file 1: Experimental Controls

1.4.1 Additional file 1: PgapA-*gfp* Expression

Colonies expressing GFP under the control of the *gapA* promoter were grown and imaged (see Figure S6). These were used as a baseline against which the fluorescence of the colonies transformed with our PaceB-*gfp* and Pacs-*gpf* plasmids could be compared. *gapA* encodes part of the glyceraldehyde 3-phosphate dehydrogenase-A complex; it is considered a housekeeping gene, and is

highly expressed by *E. coli* [6], making it a good choice for a reference. We found that the average fluorescence (in arbitrary units measured under identical conditions) observed in our *aceB* and *acs* mutant colonies was nearly half (0.467 ± 0.054) and approximately one-fourth (0.296 ± 0.048), respectively, of that of our *gapA* mutant colony. These ratios can be compared against experimental protein abundance ratios in liquid culture experiments from the literature. The consensus *aceB* and *acs* to *gapA* expression ratios found in PaxDB [7] were 0.063 and 0.006, respectively, indicating over seven-fold upregulation of *aceB* and approximately 50-fold upregulation of *acs* in our growing colonies. These findings support the hypothesis that cells in colonies upregulate genes associated with acetate utilization compared to cells growing in liquid culture. These data are summarized in Table S2.

1.4.2 Additional file 1: Anaerobic Fluorophores

Initially it was unclear whether the lack of fluorescence in the colony interiors was the result of a lack of GFP expression or a lack of the oxygen necessary for translated GFP to mature. A strain containing a plasmid expressing the flavin-based flourophore iLOV [8] under the control of the constitutive phage λ P_R promoter [9] was grown and imaged. Because *E. coli* lack the TetR repressor necessary to prevent transcription, this fluorophore should always be expressed when the cells are actively expressing protein, and, because iLOV does not require oxygen to mature, it should be visible regardless of oxygen availability. We again found rings of fluorescence within the resulting colonies (see Figure S8), indicating that the fluorescence distributions observed within these and the *aceB*, *acs*, and *gapA* colonies were likely not the result of hypoxia in the colony interior preventing GFP maturation.

1.4.3 Additional file 1: Physical Cross-sections

In order to ensure that the lack of fluorescence observed in the colony interior was not an artifact of the imaging technique, possibly arising due to scattering of the excitation or emission photons as they pass through the colony, physical cross-sections were prepared and imaged. These were made simply by cutting and moving a section of agar containing a growing colony to a microscope slide, cutting the colony and agar using a razor blade, and turning the resulting bisected colony and agar piece on their side such that the cut face of the colony and agar are in contact with the slide (see Figure S9). These slides were then imaged using a Zeiss LSM 710 confocal microscope [10]. Although this technique physically disrupts the colonies (they appear to be flatter, and some fluorescence can be smeared down into the agar), the resulting images clearly show greater fluorescence near the periphery of the colonies, confirming the results of the non-disruptive structured illumination imaging experiments (see Figure S10).

Taken together, the results of this control and the previous experiment involving colonies expressing the iLOV fluorophore (see Section 1.4.2) strongly suggest that a large portion of the colony interior is not expressing any proteins. The simplest explaination of this is that the interior cells have slipped into lag phase and are not growing. The simulated colony shows a large region of non-growing cells that make up much of the colony interior, in good agreement with this interpretation of the experimental results.

1.4.4 Additional file 1: Promoterless GFP Expression

In order to ensure that the fluorescence observed in our *aceB*, *acs*, and *gapA* experiments was not naturally occurring or merely the result of leaky transcription of the plasmid GFP, we obtained a control mutant using the same plasmid but without any promoter site. Colonies of this strain were grown and imaged in the usual manner. They showed a near complete lack of fluorescence (see Figure S11), indicating that the GFP fluorescence observed in our experiments is the result of the *aceB*, *acs*, and *gapA* promoters being active.

1.4.5 Additional file 1: Preliminary Two-Color Experiment

In order to experimentally assess whether *E. coli* colonies differentiate into the type of crossfeeding glucose- and acetate-consuming subpopulations our models predict, we attempted to construct a two-color plasmid that would enable simultaneous observation glucose-associated and acetate-associated gene expression. The plasmid was designed to include *gfp* under the control of the *aceB* promoter and the gene encoding mCherry under the control of the *ptsG* promoter. This choice to focus on *ptsG* as an indicator of glucose utilization was informed by flux balance calculations and published microarray data [11]. Optimal flux distributions for glucose- and acetate-consuming cells were computed using the *iJO1366* metabolic reconstruction with imposed crowding constraints. The microarray data was then used to compute the mRNA expression ratio for each of the 10 gene-associated reactions with the largest flux differential. The resulting data appears in Table S5. Ultimately, the gene with the largest mRNA expression ratio—*ptsG*—was chosen.

Our two-color plasmid was constructed using the PaceB-*gfp* plasmid described in the main manuscript and Table S3. Cells containing the PaceB-*gfp* plasmid were grown overnight in LB medium with 25 μg ml^{-1} kanamycin. They were then centrifuged to a pellet and plasmid DNA was harvested using a QIAprep spin miniprep kit (Qiagen). The resulting DNA was digested with the restriction enzymes BglII and BspHI (New England Biolabs). A new insert containing the promoter region for *ptsG* followed by the gene encoding mCherry was cloned into the digested region. This insert was designed to include the BglII and BspHI restriction sites near the 5' and 3' ends, respectively, the entire intergenic region upstream of *ptsG* (plus an additional 100 bases on either side), followed by the mCherry gene, and then the *ptsG* terminator region. In all, the construct was 1,323 bases, and was acquired from IDT (Integrated DNA Technologies). The modified plasmid was transformed into DH5α chemically competent cells and selected for on M9 minimal medium agar plates supplemented with 2.5 g l^{-1} glucose, trace metals, and 25 μg ml^{-1} kanamycin. Colonies were grown and imaged as described in the main manuscript.

The results of these two-color experiment strongly corroborate the existence of the crossfeeding subpopulations predicted by our model; a dome of GPF fluorescence (indicative of acetate consumption) extends across the top and sides of the colony, and mCherry fluorescence (indicative of glucose consumption) is visible throughout much of the colony interior (see Figure S11). Interestingly, although our model predicts glucose consumption to be greatest near the agar surface, the experiments appear to indicate greater consumption slightly higher up ($\sim$ 100 μm above the agar). Despite the apparent success of this experiment, we note two important observations: first, the cells we transformed and imaged grew significantly slower than those of our previous

single-color experiments (72 hours after inoculation the two-color colonies had expanded to approximately 1.2 mm in diameter; the single-color colonies had reached this approximate diameter after only 36 hours); and second, the GFP fluorescence emitted by our two-color colonies was significantly less intense than that emitted by our single-color colonies (the ratio of peak GFP fluorescence to background was approximately 10-fold lower for our two-color colonies than for our single-color colonies). We as yet have no compelling explanation for either observation, and as such we consider these results to be somewhat preliminary.

1.5 Additional file 1: Linear Radial Growth of Colonies

As the simulated colony grew, a distinct ring of fast-growing "pioneer" cells formed on the periphery. The cross-section of this ring remained relatively constant over the course of latter part of the simulation. As a toy model intended to illustrate why such a spatial distribution of growth rates would lead to a linear radial expansion of the colony, consider a cylindrical colony of constant height h, constant density ρ, and radius r of which only the outer ring of thickness δ can grow (exponentially) at specific growth rate g. For this colony we can write:

$$\begin{aligned}\frac{dM}{dt} &= \int_V \rho(\mathbf{x})g(\mathbf{x})d\mathbf{x} \\ &= g\rho h\pi\{r^2 - (r-\delta)^2\} \\ &= g\rho h\pi\{2r\delta - \delta^2\}\end{aligned} \tag{5}$$

where M represents the total mass of the colony. But we also know:

$$\begin{aligned}M &= \rho h\pi r^2 \\ \Rightarrow \frac{dM}{dt} &= 2\rho h\pi r\frac{dr}{dt}\end{aligned} \tag{6}$$

and thus:

$$\begin{aligned}\frac{dr}{dt} &= g\{\delta - \frac{\delta^2}{2r}\} \\ &\approx g\delta = \text{constant}\end{aligned} \tag{7}$$

where the last line follows because we generally assume r to be significantly larger than δ (at least in the latter parts of the simulation). Plugging in values from the simulations ($g \approx 1$ hr^{-1} and $\delta \approx 50$ μm) yields a linear expansion rate of 0.014 μm s^{-1}, in close agreement with the value observed. Although this ignores growth in the z-direction, it gives a good intuitive understanding of why the colony's radial expansion remains constant throughout much of the later portion of the simulation.

2 Additional file 1: Supporting Information Tables

Additional file 1: Table S1: Switching Rate Parameters for the Glucose-Acetate Switching Strain

Rate (s^{-1})	α_0 (s^{-1})	α_1 ($s^{-1}M^{-1}$)	α_2 ($s^{-1}M^{-1}$)	α_3 ($s^{-1}M^{-2}$)	α_4 ($s^{-1}M^{-2}$)	α_5 ($s^{-1}M^{-2}$)
$k_{\text{glc}\to\text{ace}}$	0.0	-7.8×10^{-3}	8.9×10^{-3}	0.0	2.5×10^{-2}	0.0
$k_{\text{ace}\to\text{glc}}$	2.8×10^{-4}	2.9×10^{-2}	-3.1×10^{-3}	-1.1×10^{-3}	0.0	0.0

Additional file 1: Table S2: Fluorescence and expression data

Gene	Description	Value	Units	Reference
Average colony GFP fluorescence, 5 measured colonies				
PaceB-*gfp*	average ± stdev	9.50 ± 0.57	A.U.	This study
Pacs-*gfp*	average ± stdev	6.03 ± 0.79	A.U.	This study
PgapA-*gfp*	average ± stdev	20.36 ± 1.99	A.U.	This study
aceB to *gapA* fluorescence ratio		0.467 ± 0.054	dimentionless	This study
acs to *gapA* fluorescence ratio		0.296 ± 0.048	dimentionless	This study
Consensus expression				
aceB	consensus gene product expression	809	ppm	[7]
acs	consensus gene product expression	74.1	ppm	[7]
gapA	consensus gene product expression	12772	ppm	[7]
aceB to *gapA* expression ratio		0.063	dimensionless	[7]
acs to *gapA* expression ratio		0.006	dimensionless	[7]

Additional file 1: Table S3: Bacterial strains and plasmids

Strain or Plasmid	Relevant Characteristic or Phenotype	Reference
<u>*E. coli* strain</u>		
MG1655	Wild-type *E. coli* K12, F-, λ-, *ilvG*-, *rfb*-50, *rph*-1	[12]
<u>Plasmids</u>		
pUA66	promoterless plasmid, Km^r	[12]
PaceB-*gfp*	pUA66 containing the *aceB* promoter fragment, Km^r	[12]
Pacs-*gfp*	pUA66 containing the *acs* promoter fragment, Km^r	[13]
PgapA-*gfp*	pUA66 containing the 548-bp *gapA* promoter fragment, Km^r	This study
pAM10(tet)	phage λ P_R promoter with TetR binding sites, Km^r; constitutive expression of iLOV	[9, 8]

Additional file 1: Table S4: Primers used in this study

Primer	Sequence
PgapA_XhoI_F	GGCGCGCCCTCGAGGCATCGCAGATCAAACAGTG
PgapA_BamH1_R	GGCGCGCCGGATCCGGAAAACAATGCGACCGATA
66screenF	TGGCAATTCCGACGTCTAAG
66screenR	TGTGCCCATTAACATCACCA

Additional file 1: Table S5: Predicted reaction flux differentials and mRNA expression ratios for glucose- and acetate-grown cells. Reaction flux was calculated using the FBAwMC formalism. mRNA expression ratios were calculated using microarray data from [11].

Reaction	Flux during growth on glucose (mmol gDwt^{-1} hr^{-1})	Flux during growth on acetate (mmol gDwt^{-1} hr^{-1})	Flux difference (mmol gDwt^{-1} hr^{-1})	mRNA expression ratio
GAPD_F	16.09	1.11	14.98	1.08
PGK_R	16.09	1.11	14.98	1.02
ENO	14.67	1.90	12.77	1.03
PGM	14.67	1.90	12.77	0.98
PTSG	10.05	0.00	10.05	1.15
PDH	9.15	0.00	9.15	1.04
TPI	7.20	0.47	6.73	1.06
G6PDH2r	6.04	0.00	6.04	1.04
GND	6.04	0.00	6.04	1.08
PGL	6.04	0.00	6.04	0.96

3 Additional file 1: Supporting Information Figures

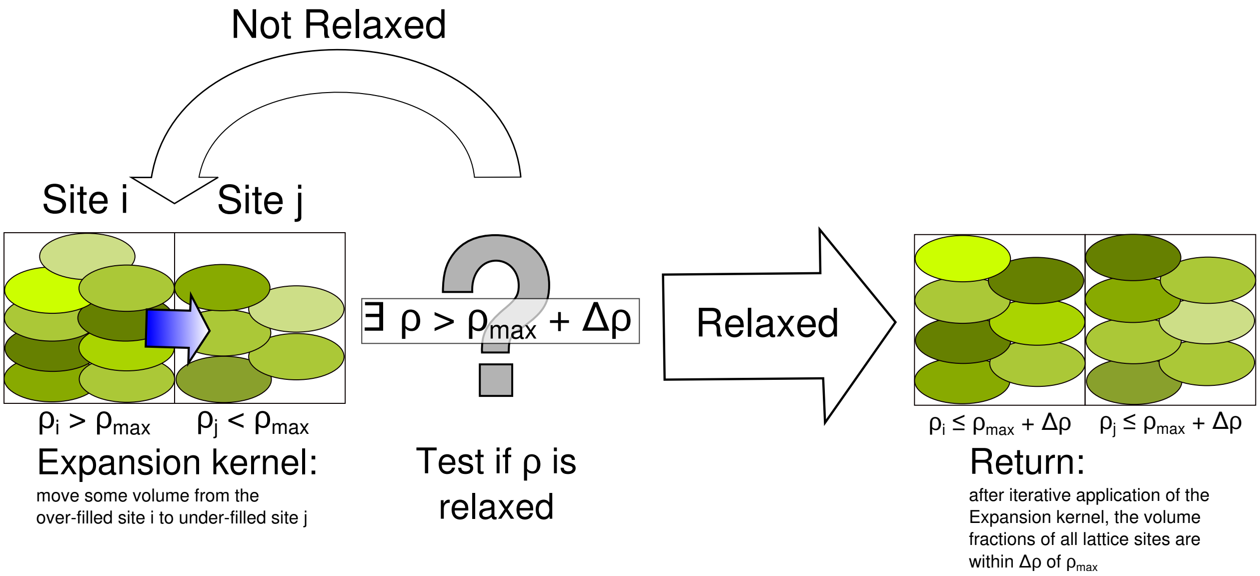

Additional file 1: Figure S1: Depiction of the Expansion kernel. The growth of the colony is slow, and so the cells are expected to have ample time to redistribute themselves such that their density remains approximately uniform at all times. This is accomplished by iteritively applying the Expansion kernel—which moves some cell volume from "over-filled" sites to neighboring sites with lower volume fractions—until no lattice site contains a volume fraction larger than some small cutoff, $\Delta\rho$ above the maximum packing fraction $\rho_{\max}$.

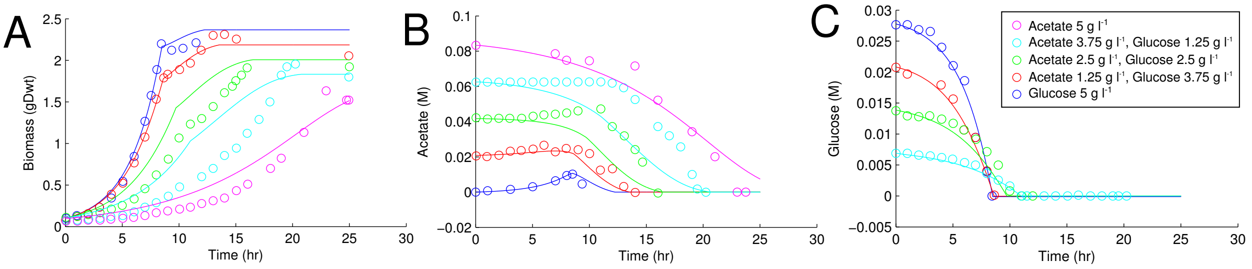

Additional file 1: Figure S2: Batch culture growth and substrate consumption by experimental and modeled *E. coli*. Data adapted from [14] are shown as circles and fits using the model described in Equation 12 of the main text are shown as lines. (A) Biomass as a function of time. (B) Acetate concentration as a function of time. (C) Glucose concentration as a function of time.

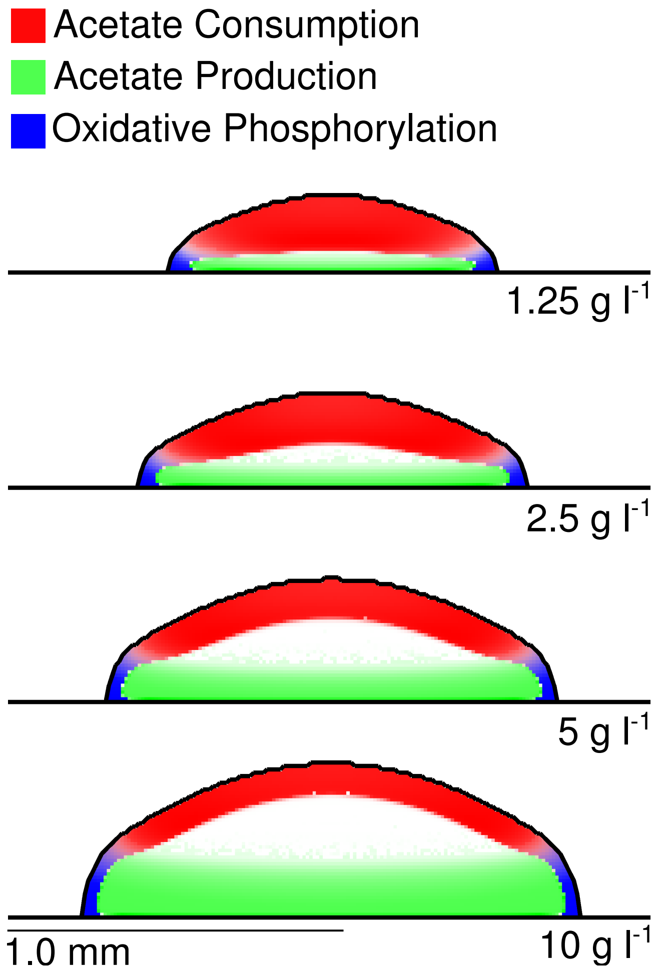

Additional file 1: Figure S3: The simulated metabolic behavior of *E. coli* colonies grown for approximately 26 hours on M9 minimal media with trace elements and varying amounts of glucose, from 1.25 g l^{-1} (top) to 10 g l^{-1} (bottom). In each case crossfeeding phenotypes comprised of acetate producers (green) and acetate consumers (red) emerge within the colony.

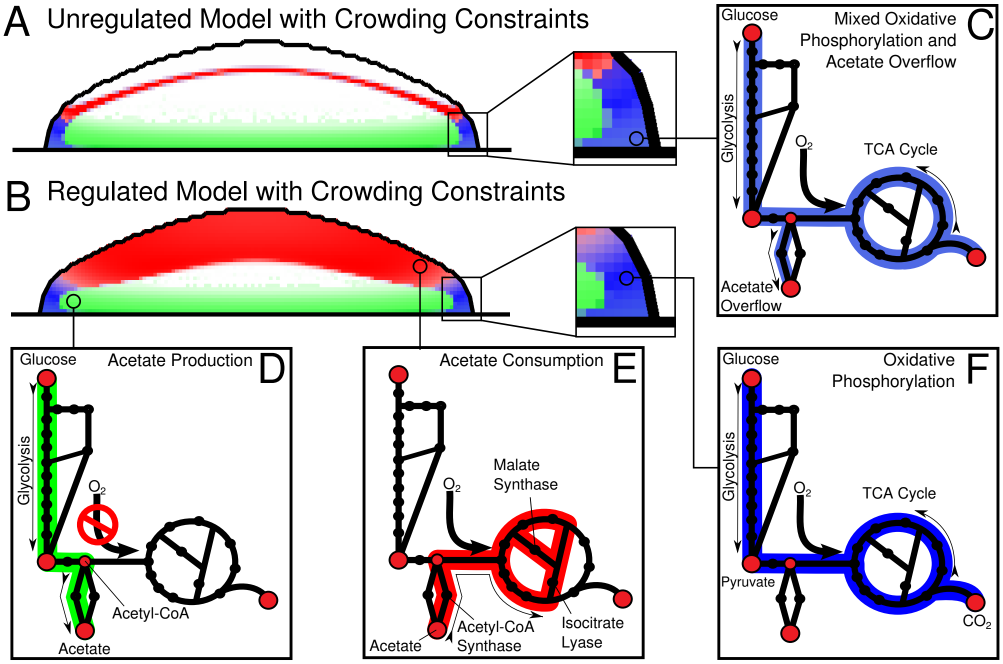

Additional file 1: Figure S4: Metabolic behaviors with crowding constraints imposed; shown are the results of 3DdFBA simulations using the FBAwMC formalism. Both "unregulated" and "regulated" models were simulated. (A) The unregulated model with crowding constraints in cross-section after 32 hours of growth. (B) The regulated model with crowding constraints in cross-section after 32 hours of growth. The addition of crowding constraints prevented the cells from engaging in simultaneous glucose and acetate utilization. (C) Cartoon of the central metabolism of cells engaged in a mixed strategy of partial oxidative phosphorylation and partial acetate overflow. The colored lines indicate flux through the metabolic network. The fastest-growing cells—those at the outermost edge nearest the agar—were driven toward this behavior because the volumetric cost of the enzymes of the TCA cycle and electron transport chain prevented exclusive oxidative phosphorylation. (D) Acetate production within both models occurred near the agar in the anoxic interior of the colony. (E) Acetate consumption occurred as a thin dome within the unregulated model and as a wider and more diffuse dome in the regulated model. (F) Pure oxidative phosphorylation occurred among some cells near the edge of the colony.

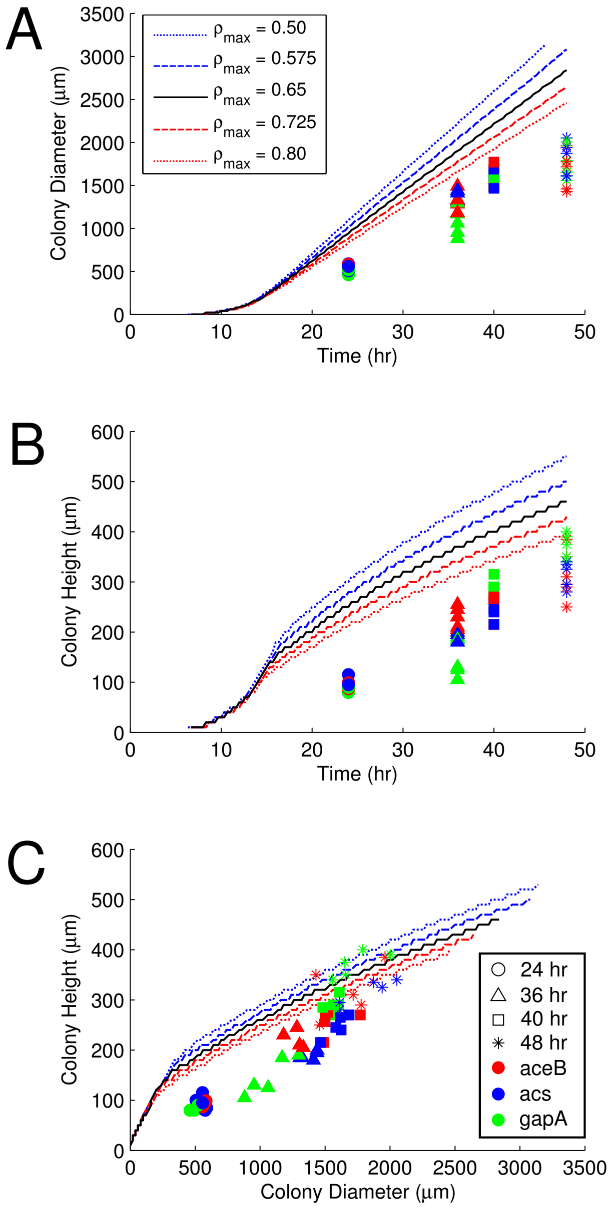

Additional file 1: Figure S5: Simulated and experimental colony heights and diameters. At 24 hours (circles), 36 hours (triangles), 40 hours (squares), and 48 hours (stars) after inoculation onto agar plates, 5 colonies of each of our fluorescent strains, PaceB-*gfp* (red), Pacs-*gfp* (blue), and PgapA-*gfp* (green), were imaged and measured. The lines indicate the height and width of modeled colonies (with regulation) over 48 hours of growth. These colonies were simulated with different values for $\rho_{\max}$ ranging from 0.50 to 0.80. The simulations presented in the main text use a value of 0.65 taken from the literature [15], and appear as the black line. The step-like features along these lines are artifacts of the discreteness of the spatial model. (A) Simulated and experimental colony diameters as a funtion of time since inoculation. (B) Simulated and experimental colony heights as a funtion of time since inoculation. (C) Colony height as a function of colony diameter. In general, lower values of $\rho_{\max}$ tend to give rise to faster-growing colonies, while higher values tend to give rise to slower-growing colonies.

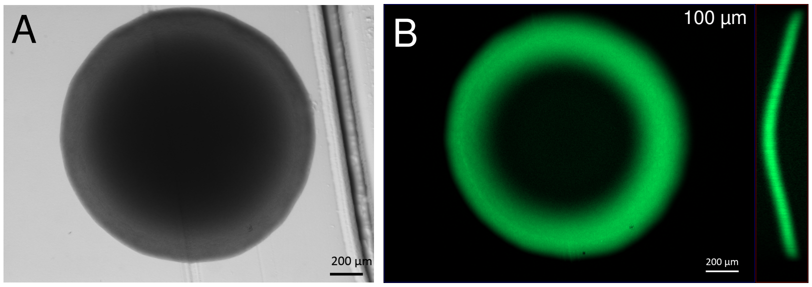

Additional file 1: Figure S6: Images of a colony expressing GFP under the control of the *gapA* promoter. (A) Brightfield image of a representative colony. (B) PgapA-*gfp* fluorescence imaged in two orthogonal planes; the first is 100 μm above and parallel to the agar surface, the second bisects the colony perpendicularly to the agar surface. *gapA* is considered a housekeeping gene in *E. coli*, making colonies like this one suitable for use as a baseline against which the fluorescence of the *aceB* and *acs* colonies could be compared. These comparisons indicated strong upregulation of *aceB* and *acs* within the growing colonies, consistent with the simulated results.

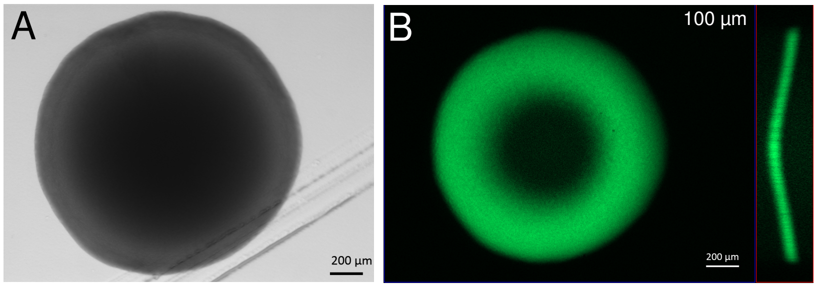

Additional file 1: Figure S7: Images of a colony expressing GFP under the control of the *acs* promoter. (A) Brightfield image of a representative colony. (B) Pacs-*gfp* fluorescence imaged in two orthogonal planes; the first is 100 μm above and parallel to the agar surface, the second bisects the colony perpendicularly to the agar surface.

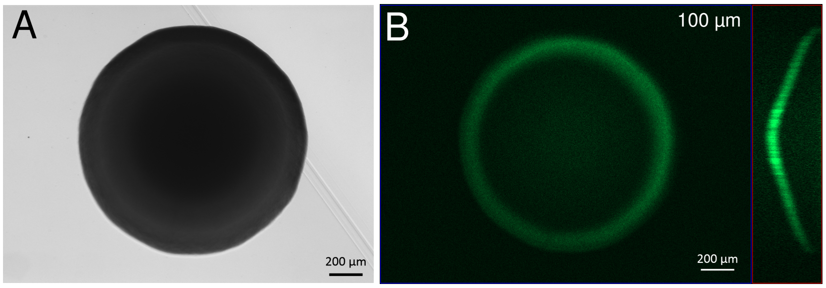

Additional file 1: Figure S8: Images of a control colony expressing the flavin-based fluorophore iLOV under the control of the constitutive phage λ P_R promoter [8, 9]. (A) Brightfield image of a representative colony. (B) iLOV fluorescence imaged in two orthogonal planes; the first is 100 μm above and parallel to the agar surface, the second bisects the colony perpendicularly to the agar surface. Although GFP is dependent on oxygen to mature, iLOV is not, meaning that the dome-like distribution of fluorescence seen in this colony and in the *aceB*, *acs*, and *gapA* colonies can not be attributed simply to hypoxia in the colony interior.

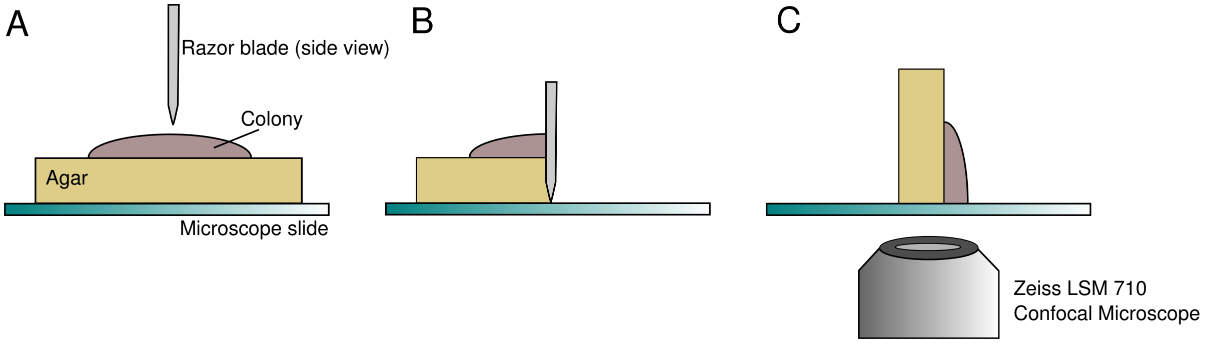

Additional file 1: Figure S9: Physical cross-sections of colonies were prepared and imaged in order to ensure that the observed distributions of fluorescence were not the result of limitations of the structured illumination imaging technique such as scattering of excitation or emission photons as they pass through the colonies. (A) A section of agar containing a growing colony is cut and removed from a petri dish and placed on a microscope slide. (B) A razor blade is used to cut the colony and agar approximately in half. (C) The remaining half of the colony and agar section is then turned such that the cut plane is in contact with the microscope slide. This plane was then imaged using a microscope.

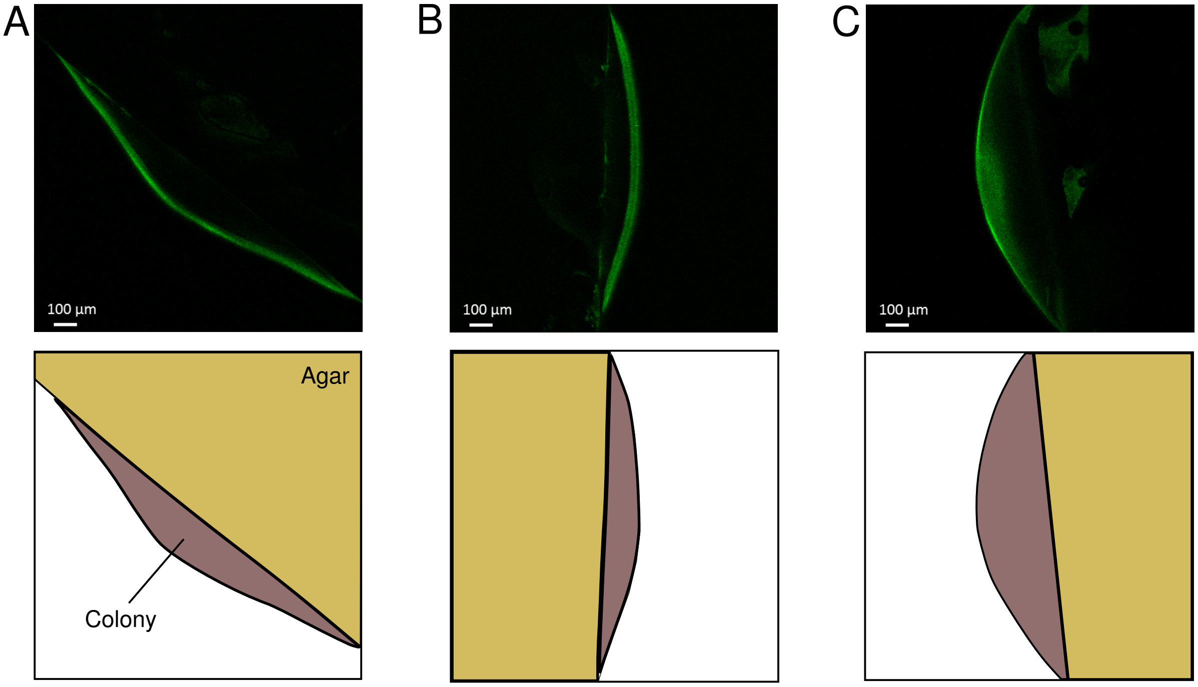

Additional file 1: Figure S10: Imaged physical cross-sections (top row) with cartoons indicating the location of the agar and the colony (bottom row). (A) A colony expressing GFP under the control of the *aceB* promoter. (B) A colony expressing GFP under the control of the *acs* promoter. (C) A colony expressing GFP under the control of the *gapA* promoter. The dome-shaped fluorescence distributions show strong agreement with the results obtained using the structured illumination technique for optical sectioning.

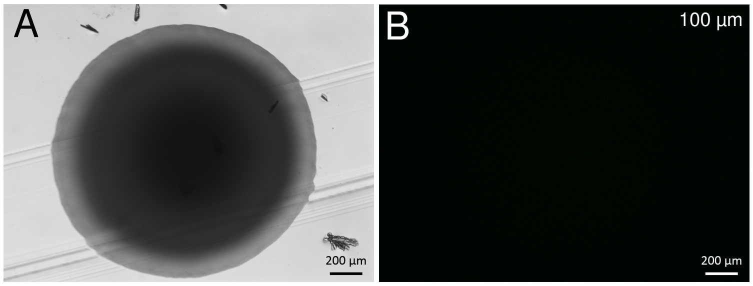

Additional file 1: Figure S11: A control colony madeup of cells transformed with the promoterless pUA66 plasmid. (A) Brightfield image. (B) GFP fluorescence of the same colony imaged at 100 μm above the agar surface. Little fluorescence is visible, indicating that the *aceB*, *acs*, and *gapA* fluorescence seen in the other experiments is the result of active transcription from their promoters rather than leaky transcription of the plasmid or background fluorescence.

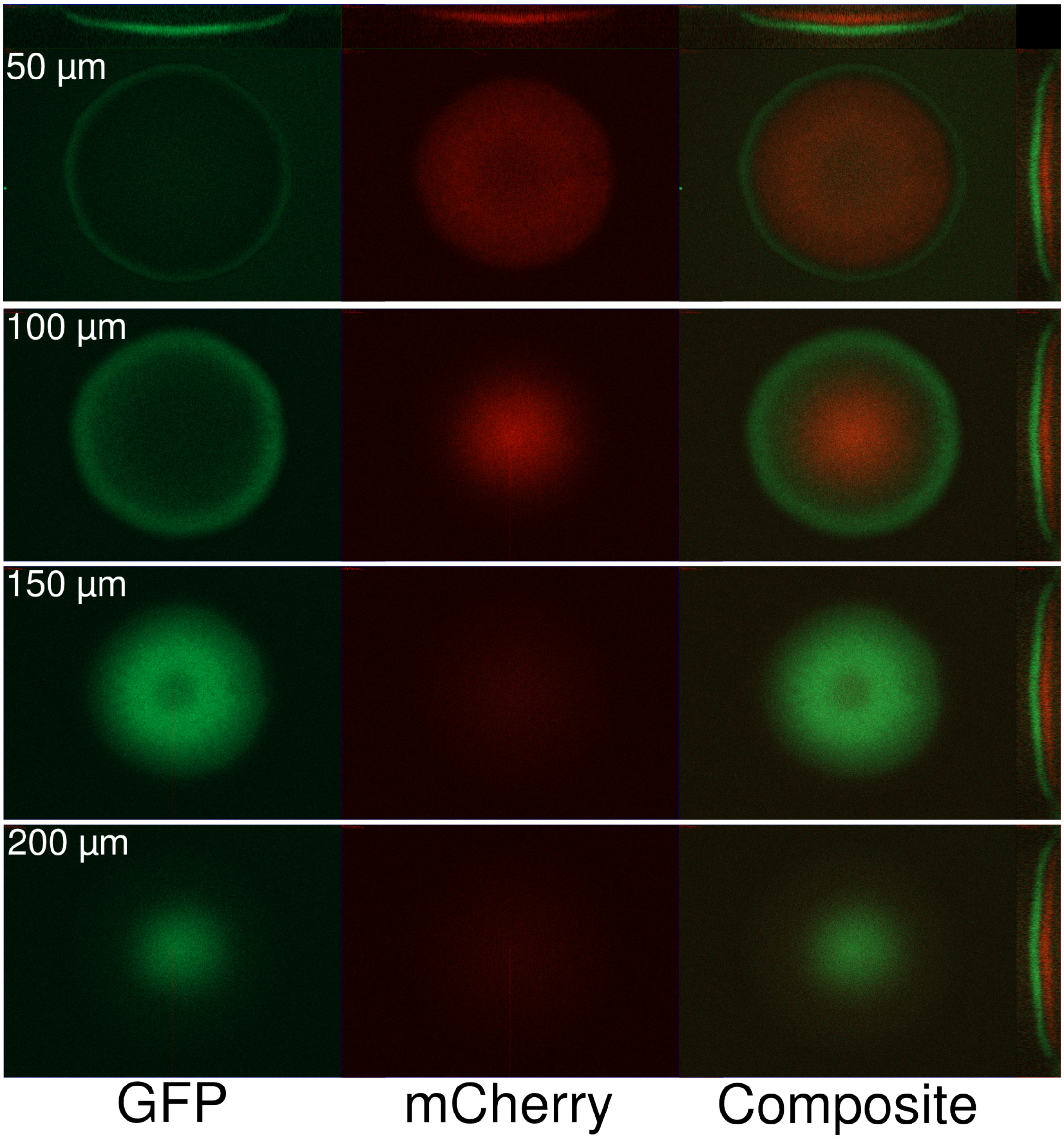

Additional file 1: Figure S12: A representative colony from our two-color experiment. Images show GFP (green) and mCherry (red) fluorescence at 50, 100, 150, and 200 μm above the agar surface.

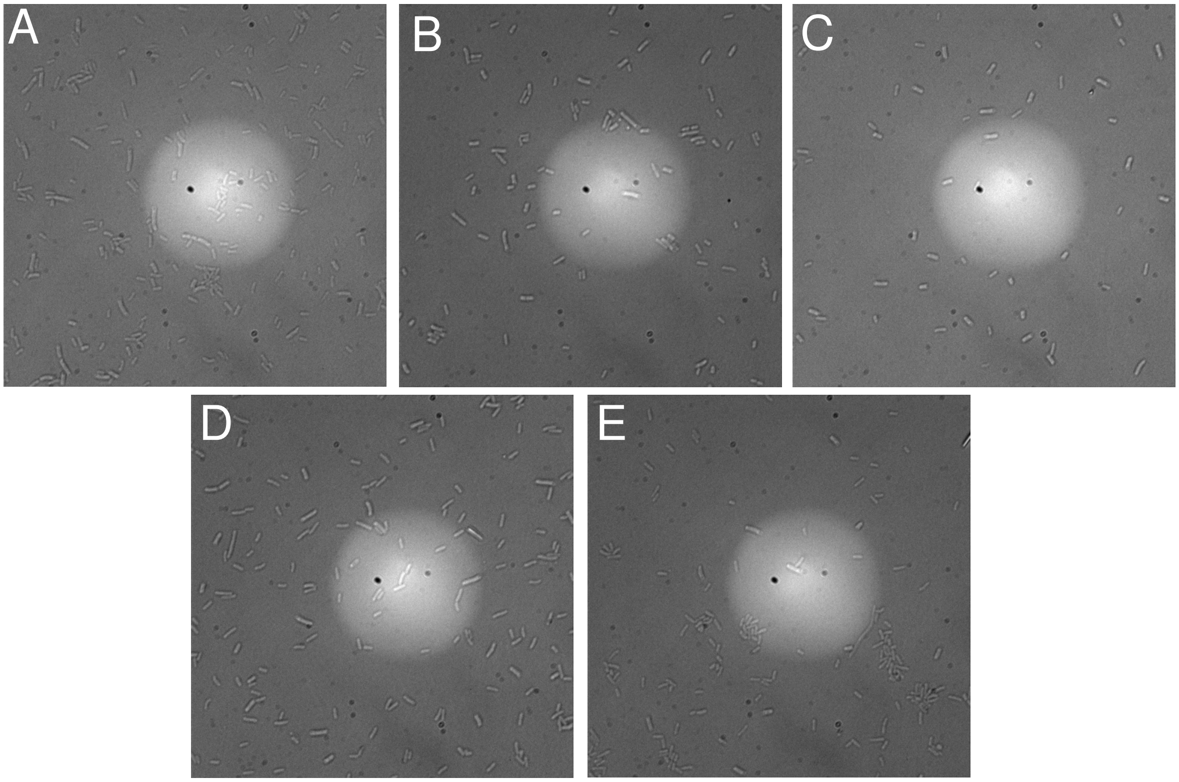

Additional file 1: Figure S13: Differential interference contrast (DIC) images of liquid culture samples of each of the strains used in this study. (A) Pacs-*gfp* strain. (B) PaceB-*gfp* strain. (C) Promoterless plasmid pUA66 strain. (D) PgapA-*gfp* strain. (E) Wild-type *E. coli* K12 MG1655

References

[1] Beuling, E.E., Van den Heuvel, J.C., Ottengraf, S.P.: Determination of biofilm diffusion coefficients using microelectrodes. Prog Biotechnol **11**, 31–38 (1996)

[2] Hallatschek, O., Hersen, P., Ramanathan, S., Nelson, D.R.: Genetic drift at expanding frontiers promotes gene segregation. Proc Natl Acad Sci U S A **104**(50), 19926–19930 (2007)

[3] Lewis, N.E., Hixson, K.K., Conrad, T.M., Lerman, J.A., Charusanti, P., Polpitiya, A.D., Adkins, J.N., Schramm, G., Purvine, S.O., Lopez-Ferrer, D., Weitz, K.K., Eils, R., König, R., Smith, R.D., Palsson, B.Ø.: Omic data from evolved *E. coli* are consistent with computed optimal growth from genome-scale models. Mol Syst Biol **6**, 390 (2010)

[4] Schellenberger, J., Que, R., Fleming, R.M., Thiele, I., Orth, J.D., Feist, A.M., Zielinski, D.C., Bordbar, A., Lewis, N.E., Rahmanian, S., *et al.*: Quantitative prediction of cellular metabolism with constraint-based models: the COBRA Toolbox v2.0. Nat Protoc **6**(9), 1290–1307 (2011)

[5] Beg, Q.K., Vazquez, A., Ernst, J., de Menezes, M.A., Bar-Joseph, Z., Barabási, A.-L., Oltvai, Z.N.: Intracellular crowding defines the mode and sequence of substrate uptake by Escherichia coli and constrains its metabolic activity. Proc Natl Acad Aci U S A **104**(31), 12663–12668 (2007)

[6] Taniguchi, Y., Choi, P.J., Li, G.-W., Chen, H., Babu, M., Hearn, J., Emili, A., Xie, X.S.: Quantifying *E. coli* Proteome and Transcriptome with Single-Molecule Sensitivity in Single Cells **329**(5991), 533–8 (2010)

[7] Wang, M., Weiss, M., Simonovic, M., Haertinger, G., Schrimpf, S.P., Hengartner, M.O., von Mering, C.: Paxdb, a database of protein abundance averages across all three domains of life. Mol Cell Proteomics **11**(8), 492–500 (2012)

[8] Chapman, S., Faulkner, C., Kaiserli, E., Garcia-Mata, C., Savenkov, E.I., Roberts, A.G., Oparka, K.J., Christie, J.M.: The photoreversible fluorescent protein iLOV outperforms GFP as a reporter of plant virus infection. Proc Natl Acad Aci U S A **105**(50), 20038–20043 (2008)

[9] Mukherjee, A., Walker, J., Weyant, K.B., Schroeder, C.M.: Characterization of flavin-based fluorescent proteins: an emerging class of fluorescent reporters. PloS One **8**(5), 64753 (2013)

[10] Sivaguru, M., Mander, L., Fried, G., Punyasena, S.W.: Capturing the surface texture and shape of pollen: a comparison of microscopy techniques. PloS One **7**(6), 39129 (2012)

[11] Faith, J.J., Driscoll, M.E., Fusaro, V.A., Cosgrove, E.J., Hayete, B., Juhn, F.S., Schneider, S.J., Gardner, T.S.: Many microbe microarrays database: uniformly normalized affymetrix compendia with structured experimental metadata. Nucleic acids research **36**(suppl 1), 866–870 (2008)

[12] Zaslaver, A., Bren, A., Ronen, M., Itzkovitz, S., Kikoin, I., Shavit, S., Liebermeister, W., Surette, M.G., Alon, U.: A comprehensive library of fluorescent transcriptional reporters for *Escherichia coli*. Nat methods **3**(8), 623–628 (2006)

[13] Nikolic, N., Barner, T., Ackermann, M.: Analysis of fluorescent reporters indicates heterogeneity in glucose uptake and utilization in clonal bacterial populations. BMC microbiol **13**(1), 258 (2013)

[14] O'Beirne, D., Hamer, G.: The utilisation of glucose/acetate mixtures by *Escherichia coli* w3110 under aerobic growth conditions. Bioprocess Eng **23**(4), 375–380 (2000)

[15] Grimson, M.J., Barker, G.C.: Continuum model for the spatiotemporal growth of bacterial colonies. Phys Rev E Stat Nonlin Soft Matter Phys **49**(2), 1680 (1994)
